# Force Dependence of Velocity and Run Length of Kinesin-1, Kinesin-2 and Kinesin-5 Family Molecular Motors

**DOI:** 10.3390/molecules24020287

**Published:** 2019-01-14

**Authors:** Si-Kao Guo, Wei-Chi Wang, Peng-Ye Wang, Ping Xie

**Affiliations:** 1 Institute of Physics, Chinese Academy of Sciences, Beijing 100190, China; sikaoguo@gmail.com (S.-K.G.); weichi@aphy.iphy.ac.cn (W.-C.W.); pywang@aphy.iphy.ac.cn (P.-Y.W.); 2 School of Physical Sciences, University of Chinese Academy of Sciences, Beijing 100049, China; 3 Songshan Lake Materials Laboratory, Dongguan 523808, Guangdong, China

**Keywords:** kinesin, processivity, velocity, chemomechanical coupling, motor protein

## Abstract

Kinesin-1, kinesin-2 and kinesin-5 are three families of a superfamily of motor proteins; which can walk processively on microtubule filaments by hydrolyzing ATP. It was experimentally shown that while the three kinesin dimers show similar feature on the force dependence of velocity, they show rather different features on the force dependence of run length. However, why the three families of kinesins show these rather different features is unclear. Here, we computationally studied the movement dynamics of the three dimers based on our proposed model. The simulated results reproduce well the available experimental data on the force dependence of velocity and run length. Moreover, the simulated results on the velocity and run length for the three dimers with altered neck linker lengths are also in quantitative agreement with the available experimental data. The studies indicate that the three families of kinesins show much similar movement mechanism and the rather different features on the force dependence of run length arise mainly from the difference in rate constants of the ATPase activity and neck linker docking. Additionally, the asymmetric (limping) movement dynamics of the three families of homodimers with and without altered neck linker lengths are studied, providing predicted results.

## 1. Introduction

Kinesins are a superfamily of motor proteins capable of walking on microtubule (MT) filaments by using the energy from ATP hydrolysis to perform diverse biological functions, including the transport of intracellular cargoes, responsibility of chromosome segregation during cell division, etc. [1]. They can generally be classified into 14 families [2]. In this paper, we focus on three families of them—kinesin-1, kinesin-2 and kinesin-5—which have been studied intensively with diverse experimental techniques.

Kinesin-1 (also called conventional kinesin) consists of two identical motor domains (heads) that are connected together by a coiled-coil stalk through their neck linkers (NLs) (each containing 14 amino acids (AAs)) [3]. Its movement dynamics is best understood and thus its movement mechanism has been studied insightfully [4]. KIF3A/B is one of the most abundant kinesin-2 family motors [5]. It is composed of two non-identical heads, KIF3A and KIF3B, which are also connected together by the coiled-coil stalk through their NLs (each containing 17 AAs). Its movement dynamics as well as the dynamics of its two homodimeric mutants, KIF3A/A and KIF3B/B, where the KIF3B head is substituted with a KIF3A head and vice versa, have been studied extensively [6,7,8,9]. Eg5, a member of kinesin-5 family, is a homotetrameric kinesin protein [10]. It is composed of two pairs of heads that are arranged anti-parallel and are connected to the opposite ends of a common stalk via their linkers (each containing 18 AAs). Each pair of heads can bind independently to an MT, so that by walking towards the plus-end, a single Eg5 motor can slide apart two antiparallel MTs that it bridges, a function that is responsible for chromosome segregation in dividing cells [11]. Both biochemical and single-molecule optical trapping experiments on dimeric Eg5 constructs, composing of a single pair of heads attached to a coiled-coil stalk, showed that the motor is minimally processive [12,13,14,15], with a run length (defined as the distance traveled by a motor per interaction with an MT) being quite short compared to kinesin-1 and kinesin-2.

Using a high-resolution single-molecule optical trapping method, the dependence of the movement dynamics such as the velocity, run length, etc., on the externally applied load acting on the coiled-coil stalk of the three families of kinesin dimers were well determined. It was shown that the three kinesin dimers show the similar feature on the force-velocity relations, with a sigmoid profile and with the velocity decreasing with the increase of the backward load [8,14,15,16,17]. However, the three dimers show rather different features on the force dependence of the run length. For example, *Drosophila* kinesin-1 shows a dramatic asymmetry of the run length with respect to the direction of the applied load: the run length under a moderate backward load is an order of magnitude longer than that under the corresponding forward load [16,17]. By contrast, kinesin-5 (Eg5) dimer exhibits a near symmetry of the run length with respect to the direction of the applied load: the run length under a backward load is nearly equal to that under the corresponding forward load [15]. More interestingly, for kinesin-2 dimers, such as mammalian KIF3A/B as well as its homodimeric constructs KIF3A/A and KIF3B/B, their run lengths drop sharply under even a small backward load relative to those under no load [8], which is contrast to cases of kinesin-1 and kinesin-5, whose run lengths under a small backward load are reduced slightly relative to those under no load. Under small forward loads, kinesin-2 run lengths are also reduced significantly compared with that under no load [18], as in the case of kinesin-1. Additionally, by altering the lengths of the NLs of the three families of kinesin dimers, it was determined experimentally that their run lengths are changed greatly. For example, extending the NLs of *Drosophila* kinesin-1 greatly reduced its run length [17,19]. Shorting the NLs of kinesin-2 (KIF3A/B) and kinesin-5 (Eg5) to 14 AAs in each head enhanced their run lengths to approach or exceed that of kinesin-1 [19,20], which has a 14-AA NL in each head.

However, why the three families of kinesin dimers show rather different features in the force dependence of the run length is unclear. Do the three dimers possess different movement mechanisms, thus giving the different features on the force dependence of the run length? If the three dimers share the similar mechanism, what are the factors that induce the different features on the force dependence of the run length? Moreover, what are the origins that shortening the NLs of the three dimers increases their run lengths? The main purpose of this paper is to clarify these unclear issues, which would have a significant implication to the movement mechanisms of the kinesin dimers. To this end, we computationally study the movement dynamics of the three dimers with the similar model to that proposed before for kinesin-1 and kinesin-2 [21] and the slightly modified model for kinesin-5. The computational data reproduce well the available experimental data on their movement dynamics such as the force dependence of the velocity and run length. The studies indicate that the three dimers show much similar movement mechanism, and the rather different features on the force dependence of the run length arise mainly from the difference in rate constants of the ATPase activity and NL docking. The computational data on the velocity and run length for the three dimers with altered NL lengths show also in quantitative agreement with the available experimental data. Additionally, the asymmetric (also called limping) movement dynamics of the three homodimers with and without altered NL lengths are studied, providing predicted results.

## 2. Methods

### 2.1. Model

**Figure 1:**
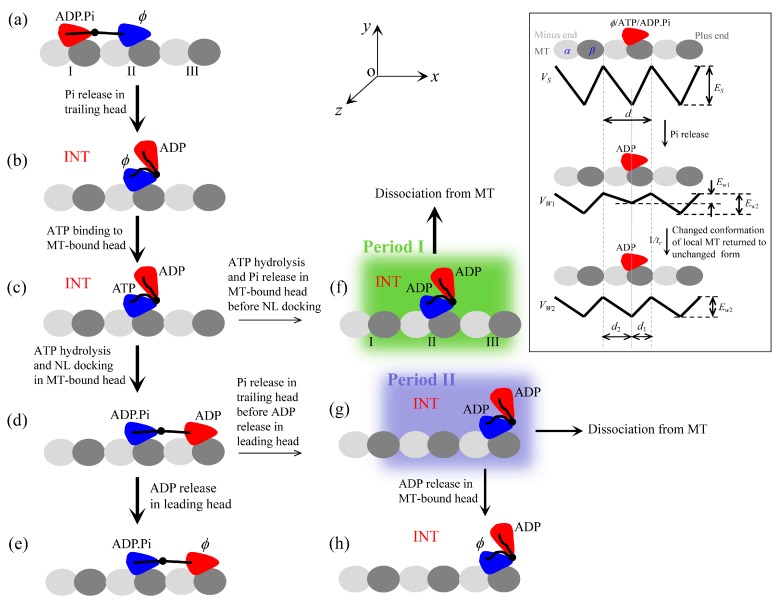
Model of a typical forward stepping of dimeric kinesin-1, kinesin-2 and kinesin-5 at low [ATP] and possible dissociation from MT. Panels inside box show potentials of a kinesin head interacting with MT along a MT filament in an ATPase cycle, with the top panel showing potential, *V*_S_, of strong interaction in ϕ, ATP or ADP.Pi states, the middle panel showing potential, *V*_W1_, of weak interaction in ADP state immediately after Pi release and the bottom panel showing potential, *V*_W2_, of weak interaction in ADP state in a period of time *t*_r_ after Pi release. (**a**) Before stepping, the trailing head in ADP.Pi state binds strongly to site I on MT while the leading head in ϕ state binds strongly to site II. (**b**) Upon Pi release, the trailing head detaches readily from site I by overcoming the very low binding energy *E*_w1_ and diffuses to the INT position relative to the MT-bound head, where the two heads have a high binding energy. (**c**) ATP binds to the MT-bound head. (**d**) After ATP hydrolysis in the MT-bound head, its NL docks rapidly, reducing the binding energy between the two heads. The detached ADP-head then diffuses to the forward site III. (**e**) ADP is released from the leading lead. (**f**) From (**c**) ATP hydrolysis and Pi can take place occasionally in the MT-bound head before its NL docks. During the period before the binding energy of the MT-bound ADP-head to the local site II changes from *E*_w1_ to *E*_w2_ (called Period I, shaded in green), the dimer can dissociate readily from MT by overcoming the very low binding energy *E*_w1_. (**g**) From (**d**) Pi can be released occasionally from the trailing head before ADP is released from the leading head. During the period before ADP is released from the MT-bound head (called Period II, shaded in blue), the dimer can dissociate from MT with a large probability by overcoming the low binding energy *E*_w2_. (**h**) From (**g**), the dimer has not dissociated from MT until ADP is released. The arrow thickness denotes the magnitude of the transition probability under low load.

The model for the processive stepping of kinesin-1, kinesin-2 and kinesin-5 is described as follows, which is modified from those presented before [4,21,22,23,24]. The model is constructed based mainly on following pieces of evidence and arguments:

(i) The kinesin head in nucleotide-free (ϕ), ATP or ADP.Pi state has a high binding energy to MT, while in ADP state has a low binding energy. Moreover, upon Pi release the head temporarily has a smaller binding energy to the local binding site (denoted by *E*_w1_) on MT than to other unperturbed binding sites (denoted by *E*_w2_ > *E*_w1_), which can be explained as follows (see Section S1 in Supplementary Information for more detail). The conformational changes of the local MT-tubulin induced by the strong interaction with ϕ, ATP- or ADP.Pi-head [25,26,27,28,29] bring about the local tubulin to have a further weaker interaction with the ADP-head than other unperturbed tubulins, as indicated by recent all-atom molecular dynamics (MD) simulations [30]. In a time of *t*_r_ (we take *t*_r_ = 10 μs, as done before [21,23,24]), the local tubulin returns to the normally unchanged conformation, with the binding energy of ADP-head to the local tubulin becoming *E*_w2_. Thus, the potential of the interaction between the kinesin head and MT along an MT filament in an ATPase cycle is illustrated inside the box of Figure 1. The mathematical equations to describe the potential are presented in Section S1 (see Supplementary Information).

(ii) For kinesin-1 head bound to MT, in ATP and ADP.Pi states the NL can be docked into its motor domain, with a docking energy denoted by *E*_NL_. The NL docking involves NL strand *β*9 forming a cover-neck bundle with strand *β*0 of the motor domain [31]. In ATP state the NL-docking efficiency or rate is much smaller than in ADP.Pi state, as argued before [21] based on the available experimental data [32,33,34,35]. In ADP and ϕ states the NL is unable to dock. For kinesin-2 head bound to MT, the nucleotide-dependent NL docking is similar to that for kinesin-1. For kinesin-5 head bound to MT, in any nucleotide state the NL can be docked, with the NL-docking rate in ADP or ϕ state being much smaller than that in ATP state and the latter being smaller than that in ADP.Pi state. As seen below, this argument for the nucleotide-dependent NL docking of the three kinesins is consistent with the available experimental data [7,36,37]. For all three kinesins, when the head is detached from MT its NL is undocked. The mathematical equations to characterize the effect of NL docking on the movement of tethered ADP-head are presented in Section S2 (see Supplementary Information).

(iii) For all three kinesins, we argue the presence of an interaction between the two heads. When the NL of the MT-bound head is undocked it has a strong interaction with the detached ADP-head, with the binding energy denoted by *E*_I1_, and the NL docking in the MT-bound head weakens the interaction, with the binding energy denoted by *E*_I2_ (< *E*_I1_). The argument is consistent with the preliminary all-atom MD simulation results (Shi et al., unpublished). The mathematical equations to describe the interaction between the two heads are presented in Section S3 (see Supplementary Information).

It is noticed that with arguments (ii) and (iii) the biochemical data of Hackney [37] for kinesin-1, showing that after mixture of the dimer with both heads bound by ADP with MT only half fraction of ADP molecules are released and addition of ATP results in release of other half fraction of ADP molecules, are understandable readily. The similar biochemical data for kinesin-2 [7] can also be explained well. The biochemical data showing that in ϕ state kinesin-5 resides in a two-head-bound (2HB) state [36] are consistent with arguments (ii) and (iii). The biochemical data showing that in ADP state kinesin-5 dimer has a much slower MT unbinding rate than kinesin-5 monomer [36] can also be explained well based on arguments (ii) and (iii). The biochemical data for kinesin-5, showing that addition of non-hydrolyzing ATP analogs leads to about 2-fold slower mant-ADP release than addition of ATP [36], can also be explained with arguments (ii) and (iii) (see Section 2.4).

Based on the above evidence and/or arguments, a typical forward stepping of the kinesin dimer at low ATP concentration ([ATP]) is illustrated in Figure 1. We begin with the trailing head in ADP.Pi state binding strongly to site I on MT while the leading head in ϕ state binding strongly to site II (Figure 1a). After Pi release (with rate constant *k*_Pi_) from the trailing head, the ADP-head detaches readily from site I by overcoming the very low binding energy *E*_w1_ and diffuses rapidly to the intermediate (INT) position relative to the MT-bound head, where the two heads bind strongly together (Figure 1b). After ATP binding to the ϕ-head (with the second-order binding rate *k*_b_) (Figure 1c), followed by ATP hydrolysis (with rate constant *k*_H_), NL docking takes place rapidly (with rate constant *k*_NL_), reducing the binding energy between the two heads. The detached ADP-head then diffuses rapidly to the forward site III (Figure 1d). Note here that an energy barrier *E*_NL_ arising from the NL docking in the MT-bound head leads to the biased diffusion of the detached ADP-head to the forward site III by preventing it from moving backward to the backward site I. After the ADP-head binding to site III, ADP is released (with a rate constant *k*_D_) (Figure 1e).

In INT state (Figure 1c), ATP hydrolysis and Pi release can also take place occasionally in the MT-bound head before its NL docks (Figure 1f). In Figure 1f during the period before the binding energy of the MT-bound ADP-head to the local site II changes from *E*_w1_ to *E*_w2_ (called Period I), the dimer can easily dissociate from MT by overcoming the very low *E*_w1_. In Figure 1d, Pi can also be released occasionally from the trailing head before ADP is released from the leading head (Figure 1g). In Figure 1g during the period before ADP is released from the MT-bound head (called Period II), the dimer can also dissociate from MT with a large probability by overcoming weak *E*_w2_. If the dimer has not dissociated until ADP is released (Figure 1h), the system becomes the same as Figure 1b except that a forward step of the dimer was made.

It should be mentioned that in Figure 1 we show only main state transitions and possible MT unbinding in a mechanochemical coupling cycle. Besides, other minor transitions can also occur occasionally. For example, for kinesin-5, during transition from Figure 1b to c, before ATP binding to the ϕ-head its NL can also dock occasionally. The detached ADP-head then binds to the forward site III. After ATP binding to the trailing ϕ-head and ATP hydrolysis, the system becomes the state of either Figure 1d if ADP has not been released from the leading head or Figure 1e if ADP has been released from the leading head. For all three kinesins, after NL docking in the MT-bound head in INT state the detached ADP-head can also possibly diffuse to and bind the backward site on MT, giving a futile mechanochemical coupling cycle. ATP binding, ATP hydrolysis and Pi release can also occur occasionally in the leading head, resulting in a futile mechanochemical coupling cycle or a backward step.

Based on the model (Figure 1), the equations for the mechanical stepping and MT unbinding are presented in Section S5 (see Supplementary Information). The details of the simulation procedure of the processive movement by combining the mechanical stepping with continuous ATPase activity are described in Section S6 (see Supplementary Information). In this work, we take parameter values of *Drosophila* kinesin-1 as an example to study movement dynamics of kinesin-1, those of mammalian KIF3A/B, KIF3A/A and KIF3B/B as an example to study movement dynamics of kinesin-2, and those of Eg5 as an example to study movement dynamics of kinesin-5. Their values are chosen as follows.

### 2.2. Parameter Values of Kinesin-1

First, consider kinesin-1. We take *E*_w1_ = 19.8*k_B_T* and *E*_w2_ = 39.5*k_B_T*. Since *E*_w1_ and *E*_w2_ have sensitive effects on the run length, their values are adjusted here to make the run length under no load be close to the available experimental data. We take NL-docking energy *E*_NL_ = 6*k_B_T*. Since *E*_NL_ has a sensitive effect on the reduction of velocity under a large backward load relative to that under no load, its value is adjusted here to make the reduction of velocity under a given large backward load (e.g., −5 pN) relative to that under no load be close to the available experimental data. Provided that *E*_I1_ > 40*k_B_T* and *E*_I2_ < 20*k_B_T*, varying values of *E*_I1_ and *E*_I2_ has nearly no effect on the results (also applicable to kinesin-2 and kinesin-5). As done before [21], we take NL-docking rate *k*_NL_ = 800 s^−1^ when the MT-bound head in INT state is in ADP.Pi state, *k*_NL_ ≤ 1 s^−1^ in ATP state and *k*_NL_ = 0 in ϕ or ADP state, which are based on the available experimental results [32,33,34,35,38]. Under a backward load, considering that in INT state the NL of the MT-bound head would be stretched backward, the NL-docking rate would be reduced. Thus, it is expected that under a backward load the NL-docking rates should be reduced by λ-fold (*λ* ≥ 1) relative to those given above under no or forward load. We have checked that provided *λ* ≤ 1.2, varying *λ* has nearly no effect on the simulated results. For the calculation, we take *λ* = 1.2 here.

Based on the available experimental data indicating that the tension on the NL has nearly no effect on ATPase rate of the kinesin-1 dimer [39], the external force affects the affinity of ADP for the head [40,41] and ADP release is a non-rate-limiting step of ATP turnover [42], we take into account the following considerations. The rate constants of ATP binding (*k*_b_) and hydrolysis (*k*_H_) are independent of the tension on the NL and the orientation or pointing direction of the NL. The rate constant of Pi release (the rate-limiting step of ATP turnover) is independent of the tension on the NL but is dependent of the pointing direction of the NL. The ADP-release rate (*k*_D_) is dependent of the tension on the NL. We take *k*_b_ = 2 μM·s^−1^ and *k*_H_ = 350 s^−1^, consistent with the biochemical data [42,43]. When the NL points forward the head has a much larger rate constant of Pi release than when the NL does not point forward. This is understandable, because when pointing forward the NL can interact with the head, enhancing the rate constant of Pi release, as the experimental data showed that deletion of NL, i.e., with no interaction between the NL and the head, reduces the ATPase rate significantly whereas has no effect on ADP-release rate [44]. Hence, in 2HB state the Pi-release rate, $k_{\mathrm{Pi}}^{\left(L\right)}$, of the leading head is much smaller than that, $k_{\mathrm{Pi}}^{\left(T\right)}$ ($k_{\mathrm{Pi}}^{\left(T\right)}$ = *k*_Pi_), of the trailing head. In INT state, the NL of the MT-bound head is considered to point forward when it is stretched to a length *l*_NL_ > 2.8 nm under a forward load and thus the Pi-release rate of the MT-bound head is equal to *k*_Pi_, otherwise the Pi-release rate is equal to $k_{\mathrm{Pi}}^{\left(L\right)}$. In the calculation, we take $k_{\mathrm{Pi}}^{\left(L\right)}$ = $k_{\mathrm{Pi}}/\rho$, with ρ = 80, and *k*_Pi_ = 160 s^−1^ that is consistent with the experimental data [42,43]. Since for kinesin-1 with the NL of each head having 14 AAs the internally stretched force on the NLs has a large value of about 30 pN in 2HB state (see Figure S1, at extension of 4.1 nm), we take ADP-release rate, $k_{D}^{\left(T\right)}$ = $k_{D}^{\left(L\right)}/\sigma$ ($k_{D}^{\left(L\right)}$ = *k*_D_), with σ = 2.3 (in the small range of the external load, we approximate that *k*_D_ and σ are constant). In INT state, the rate of the ADP release from the MT-bound head is also taken as $k_{D}/\sigma$. We take *k*_D_ = 350 s^−1^, consistent with the experimental data [42,43]. When ADP-head is not bound to MT, *k*_D_ = 0. The above parameter values for kinesin-1 are listed in Table 1.

### 2.3. Parameter Values of Kinesin-2

Second, consider kinesin-2. Kinesin-2 is composed of two different heads KIF3A and KIF3B. For KIF3A head, we take *E*_w1_ ≤ 18*k_B_T*, *E*_w2_ = 40.8*k_B_T* and *E*_NL_ = 9.3*k_B_T*. For KIF3B head, we take *E*_w1_ ≤ 18*k_B_T*, *E*_w2_ = 39.5*k_B_T* and *E*_NL_ = 9.2*k_B_T*. These values of *E*_w1,_ *E*_w2_ and *E*_NL_ are adjusted here, as done for kinesin-1 (see above). As for kinesin-1, we have the same consideration of nucleotide-dependent NL docking for kinesin-2, but with values of *k*_NL_ for kinesin-2 being 1.5-fold larger than those for kinesin-1. For both KIF3A and KIF3B heads, under no or forward load we take *k*_NL_ = 1200 s^−1^ in ADP.Pi state, *k*_NL_ ≤ 1.5 s^−1^ in ATP state and *k*_NL_ = 0 in ϕ or ADP state. Under a backward load, values of *k*_NL_ are reduced by λ-fold relative to those given above, with λ = 4 and 2 for KIF3A and KIF3B heads, respectively.

As for kinesin-1, we have the same consideration for kinesin-2 on the dependence of rate constants of ATP binding (*k*_b_), ATP hydrolysis (*k*_H_), Pi release (*k*_Pi_) and ADP release (*k*_D_) upon the tension and orientation direction of the NL. For both KIF3A and KIF3B heads, we take *k*_b_ = 2 μM·s^−1^ and *k*_H_ = 350 s^−1^. In 2HB state, the rate constant of Pi release in the leading head, $k_{\mathrm{Pi}}^{\left(L\right)}$, is much smaller than that in the trailing head, $k_{\mathrm{Pi}}^{\left(T\right)}$ ($k_{\mathrm{Pi}}^{\left(T\right)}$ = *k*_Pi_). In INT state, the Pi-release rate of the MT-bound head is equal to *k*_Pi_ when its NL is stretched to a length *l*_NL_ > 2.8 nm under a forward load, otherwise the Pi-release rate is equal to $k_{\mathrm{Pi}}^{\left(L\right)}$. We take $k_{\mathrm{Pi}}^{\left(L\right)}$ = $k_{\mathrm{Pi}}/\rho$, with *k*_Pi_ = 150 s^−1^ and ρ = 17 for KIF3A head, and *k*_Pi_ = 170 s^−1^ and ρ = 40 for KIF3B head. Since for kinesin-2 with the NL of each head having 17 AAs the internally stretched force on the NLs is very small in 2HB state (see Figure S2, at extension of 4.1 nm), we take $k_{D}^{\left(T\right)}$ = $k_{D}^{\left(L\right)}$ = *k*_D_. In INT state, the rate of the ADP release from the MT-bound head is also equal to *k*_D_. To be consistent with the biochemical data showing that the ADP-release rate for homodimeric KIF3A/A and KIF3B/B is about 80–90 s^−1^ [9], we take *k*_D_ = 90 s^−1^ for both KIF3A and KIF3B heads. When ADP-head is not bound to MT, *k*_D_ = 0. The above parameter values for KIF3A and KIF3B heads are listed in Table 1.

### 2.4. Parameter Values of Kinesin-5

Third, consider kinesin-5. We take *E*_w1_ ≤ 18*k_B_T*, *E*_w2_ = 36.5*k_B_T* and *E*_NL_ = 7.5*k_B_T*. These values of *E*_w1,_ *E*_w2_ and *E*_NL_ are adjusted here, as done for kinesin-1 and kinesin-2 (see above). As for kinesin-1 and kinesin-2, we have the same consideration for kinesin-5 on the dependence of *k*_b_, *k*_H_, *k*_Pi_ and *k*_D_ on the tension and orientation direction of the NL. In 2HB state, we take $k_{\mathrm{Pi}}^{\left(L\right)}$ = $k_{\mathrm{Pi}}^{\left(T\right)}/\rho$ = $k_{\mathrm{Pi}}/\rho$, with ρ = 80, as done above for kinesin-1. In INT state, the Pi-release rate of the MT-bound head is equal to *k*_Pi_ when its NL is stretched to a length *l*_NL_ > 2.8 nm under a forward load, otherwise the Pi-release rate is equal to $k_{\mathrm{Pi}}^{\left(L\right)}$. Based on the biochemical data showing that ATP hydrolysis is fast [36], we take *k*_H_ = 350 s^−1^, as for kinesin-1 and kinesin-2. Since for kinesin-5 with the NL of each head having 18 AAs the internally stretched force on the NLs is almost zero in 2HB state (see Figure S3, at extension of 4.1 nm), we take ADP-release rate, $k_{D}^{\left(T\right)}$ = $k_{D}^{\left(L\right)}$ = *k*_D_. In INT state, the rate of the ADP release from the MT-bound head is also equal to *k*_D_. To be consistent with the biochemical data showing that ATP-triggered mant-ADP release with a rate of about 50 s^−1^ and non-hydrolyzing ATP analogs lead to 2-fold slower mant-ADP release [36] and considering *k*_H_ having a value (350 s^−1^, see above) much larger than 50 s^−1^, we take *k*_D_ = 50 s^−1^ and *k*_NL_ = 50 s^−1^ when the MT-bound head in INT state is in ATP state. We take *k*_NL_ = 300 s^−1^ when the MT-bound head is in ADP.Pi state, which is consistent with the biochemical data indicating that kinesin-5 has a slower NL-docking rate than kinesin-1 [45]. We take *k*_NL_ ≤ 0.1 s^−1^ when the MT-bound head is in ϕ or ADP state. Under a backward load, we take values of *k*_NL_ to be reduced by λ-fold relative to those given above under no or forward load, with λ = 4. Based on the biochemical data showing that the ATP-turnover rate is about 12.9 ± 1.9 s^−1^ [36] and considering that Pi release is the rate-limiting step, we take *k*_Pi_ = 15 s^−1^. In addition, we take *k*_b_ = 0.5 μM·s^−1^. The above parameter values for kinesin-5 are listed in Table 1.

## 3. Results and Discussion

As in the single-molecule optical trapping experiments [8,14,15,16,17], we consider an externally applied load acting on a micrometer-sized bead attached to the coiled-coil stalk of the dimer (see Section S5 in Supplementary Information). The longitudinal component of the load or force, *F_x_*, is defined to have negative and positive values when it resists and assists the forward movement, respectively (see Section S5 in Supplementary Information).

### 3.1. Velocity of kinesin Kimers

**Figure 2:**
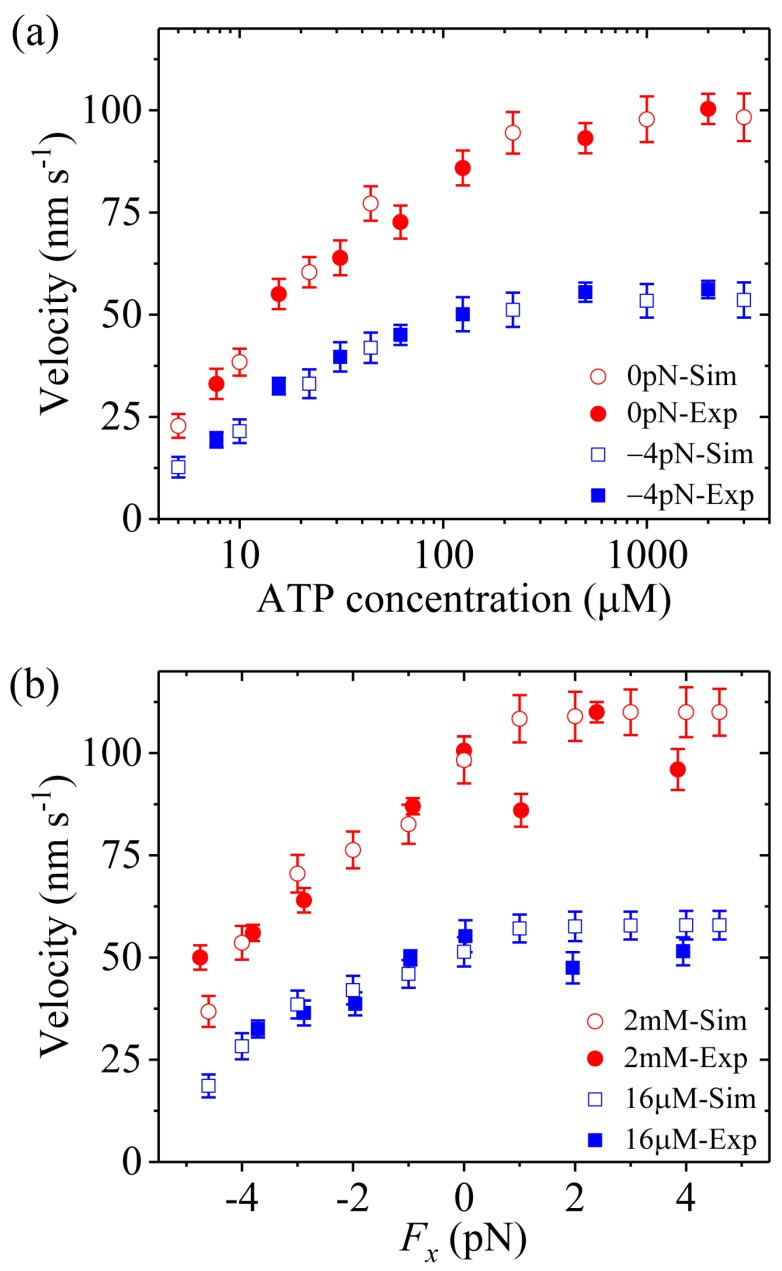
Kinesin-5 velocity as a function of [ATP] and force. Unfilled circles and squares are simulated data, while filled circles and squares are experimental data taken from Valentine et al. [14]. (**a**) Velocity *versus* [ATP] at *F_x_* = 0 and *F_x_* = –4 pN. (**b**) Velocity *versus F_x_* at [ATP] = 2 mM and [ATP] = 16 μM.

First, we study kinesin-5. Figure 2a,b shows simulated results of the velocity *versus* [ATP] at different values of the applied load and *versus* the applied load at different values of [ATP], respectively. For comparison, the corresponding single-molecule data of Valentine et al. [14] are also plotted in Figure 2. It is seen that the simulated data show good agreement with the experimental data.

**Figure 3:**
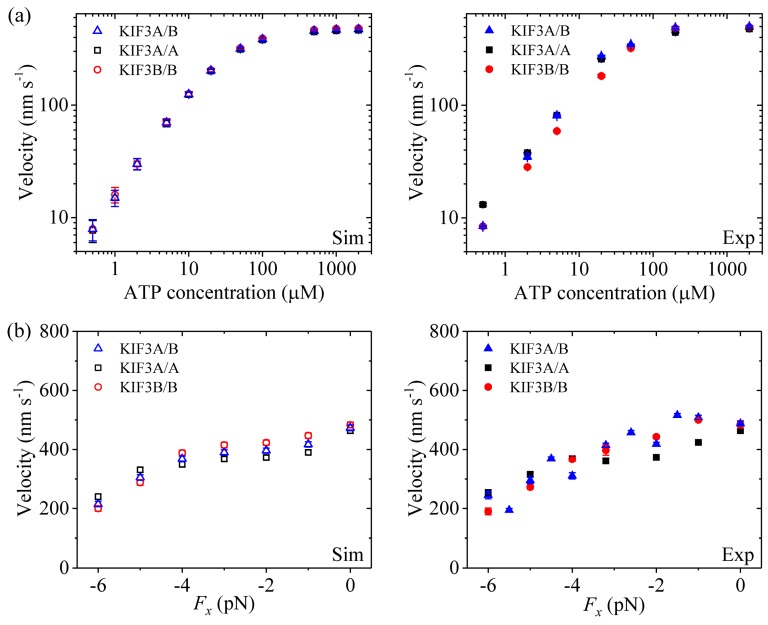
Kinesin-2 velocity as a function of [ATP] and force. Left panels show simulated data, while right panels show experimental data taken from Andreasson et al. [8]. (**a**) KIF3A/B, KIF3A/A and KIF3B/B velocity *versus* [ATP] at *F_x_* = 0. (**b**) KIF3A/B, KIF3A/A and KIF3B/B velocity *versus F_x_* at [ATP] = 2 mM.

Second, we study kinesin-2. Figure 3a (left panel) shows simulated results of the velocity *versus* [ATP] under no applied load. Figure 3b (left panel) shows simulated results of the velocity *versus* the applied load at saturating [ATP] (2 mM). For comparisons, the single-molecule data of Andreasson et al. [8] are shown in right panels of Figure 3. It is seen that the simulated and experimental data are in good agreement with each other.

**Figure 4:**
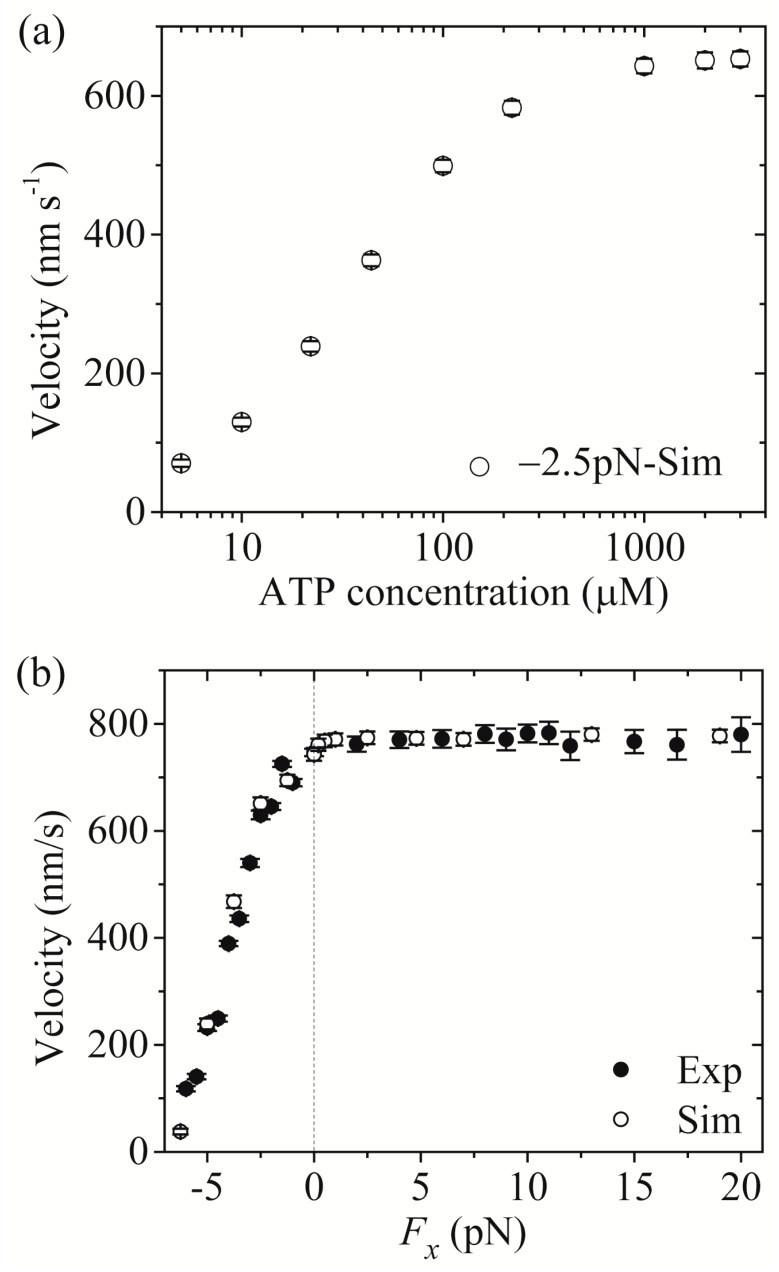
Kinesin-1 velocity as a function of [ATP] and force. Unfilled circles are simulated data, while filled circles are experimental data taken from Andreasson et al. [17]. (**a**) Velocity *versus* [ATP] at *F_x_* = −2.5 pN. (**b**) Velocity *versus F_x_* at [ATP] = 2 mM.

Third, we study kinesin-1. The results simulated with λ = 1 were presented before [21]. Here, we use λ = 1.2 (see Table 1) to make simulations. Figure 4a,b shows simulated results of the velocity *versus* [ATP] under a backward load (−2.5 pN) and *versus* the applied load at saturating [ATP] (2 mM), respectively. For comparison, the single-molecule data of Andreasson et al. [17] are also shown in Figure 4b. From Figure 4b we see that the simulated and experimental data are in good agreement with each other.

From Figure 2a, Figure 3a and Figure 4a we see that for all of the three families of kinesin dimers the dependence of the velocity on [ATP] follows the Michaelis-Menten relation. From Figure 2b, Figure 3b and Figure 4b we see that for all of the three families of kinesin dimers, the dependence of the velocity on force has a sigmoid form and the velocity decreases as the backward force increases. However, the velocities of kinesin-5 and kinesin-2 are less affected by the backward force than the velocity of kinesin-1, which arises mainly from the fact that kinesin-5 and kinesin-2 have larger NL-docking energy *E*_NL_ than kinesin-1 (see Table 1).

Taken together, since the three families of kinesin dimers show much similar mechanism of the processive movement, they exhibit the similar feature on the dependence of the velocity upon [ATP] and upon the external force. However, since kinesin-5 and kinesin-2 have larger NL-docking energy than kinesin-1, the velocities of kinesin-5 and kinesin-2 are less affected by the backward force than the velocity of kinesin-1.

### 3.2. Processivity of Kinesin Dimers

**Figure 5:**
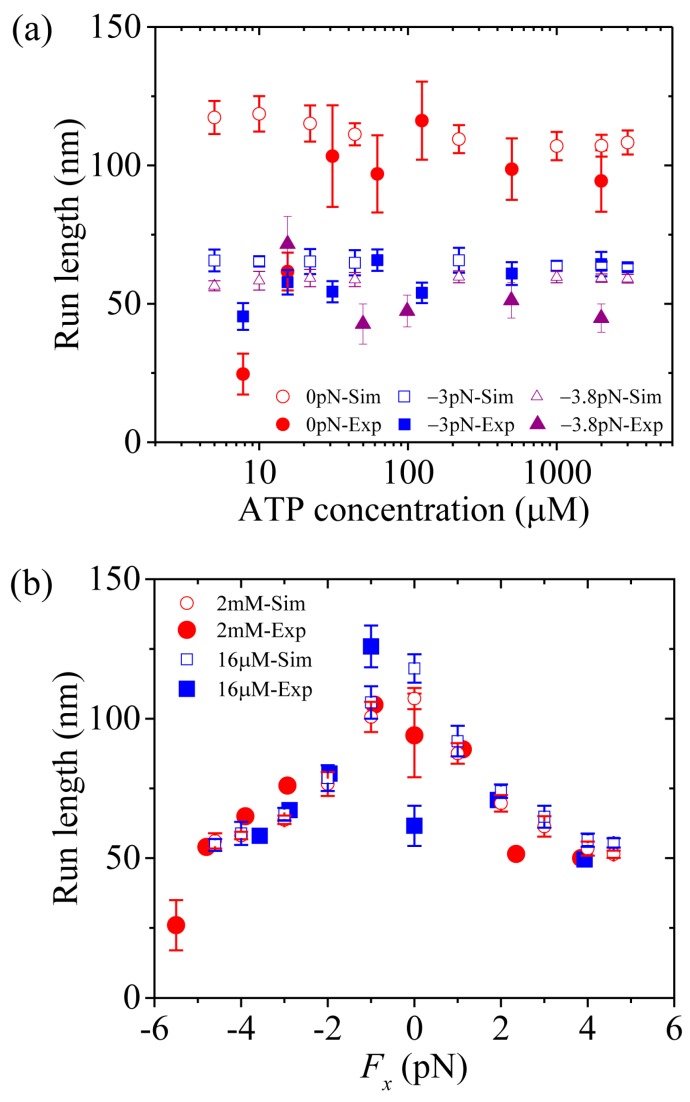
Kinesin-5 run length as a function of [ATP] and force. Unfilled circles, squares and triangles are simulated data, while filled circles, squares and triangles are experimental data taken from Valentine and Block [15]. (**a**) Run length *versus* [ATP] at *F_x_* = 0, *F_x_* = –3 pN and *F_x_* = –3.8 pN. (**b**) Run length *versus F_x_* at [ATP] = 2 mM and [ATP] = 16 μM.

First, we study kinesin-5. Figure 5a,b shows simulated results of the run length *versus* [ATP] at different values of the applied load and *versus* the applied load at different values of [ATP], respectively. For comparisons, the corresponding single-molecule data of Valentine and Block [15] are also plotted in Figure 5. It is seen that the simulated and experimental data are in quantitative agreement with each other.

**Figure 6:**
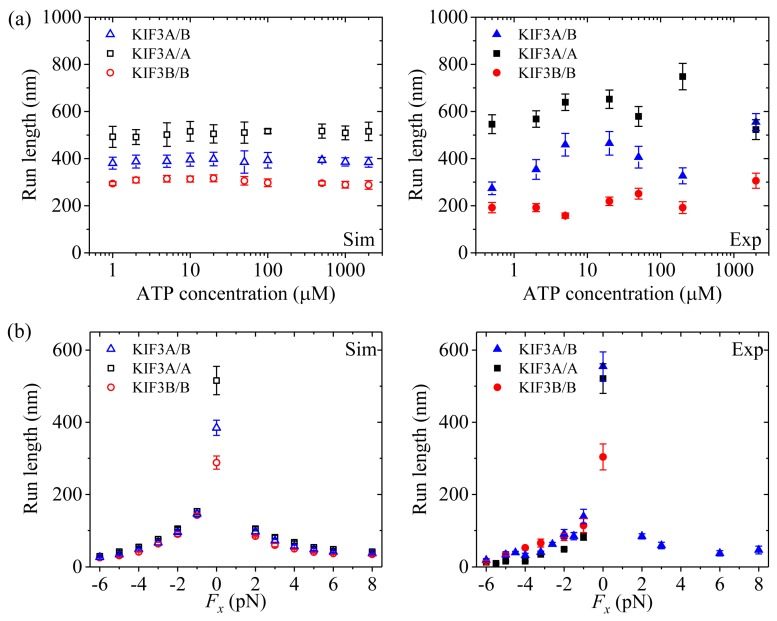
Kinesin-2 run length as a function of [ATP] and force. Left panels show simulated data, while right panels show experimental data taken from Andreasson et al. [8,18]. (**a**) KIF3A/B, KIF3A/A and KIF3B/B run length *versus* [ATP] at *F_x_* = 0. (**b**) KIF3A/B, KIF3A/A and KIF3B/B run length *versus F_x_* at [ATP] = 2 mM.

Second, we study kinesin-2. Figure 6a (left panel) shows simulated results of the run length *versus* [ATP] under no applied load. Figure 6b (left panel) shows simulated results of the run length *versus* the applied load at saturating [ATP] (2 mM). For comparisons, the corresponding single-molecule data of Andreasson et al. [8,18] are shown in right panels of Figure 6. It is seen that the simulated and experimental data show consistent with each other.

**Figure 7:**
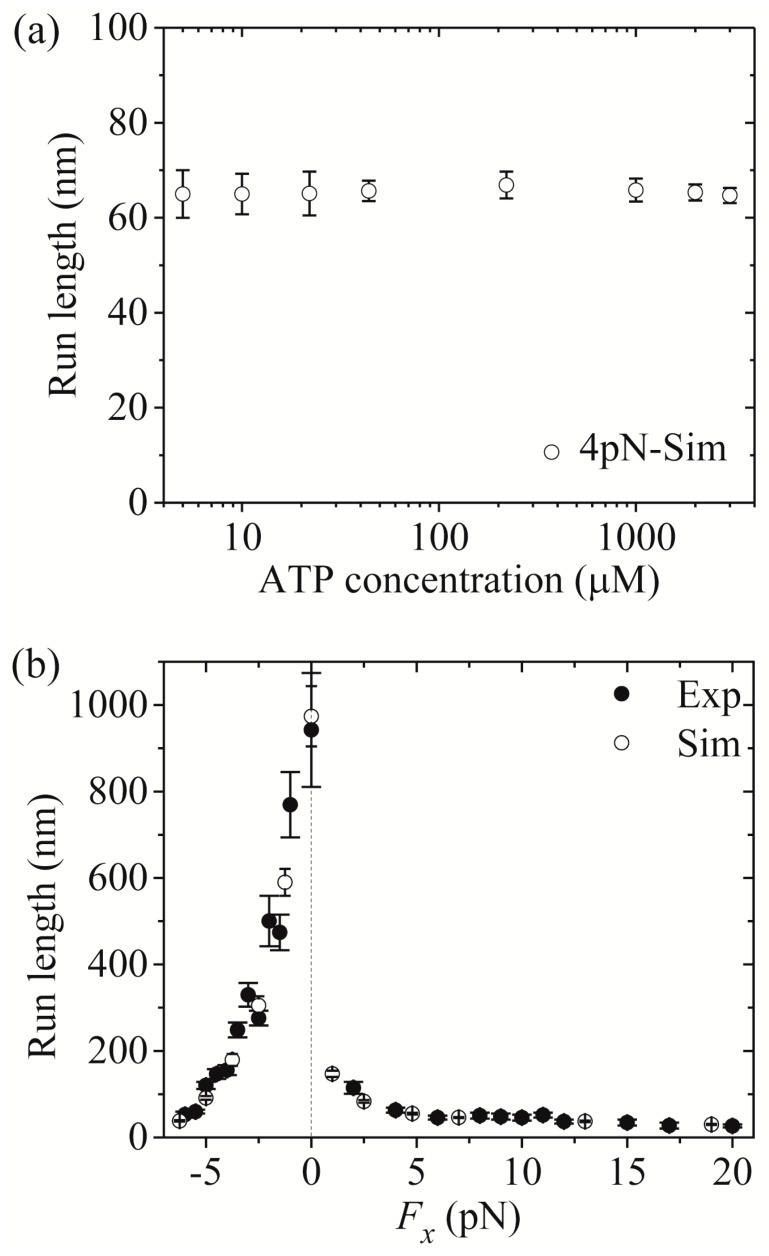
Kinesin-1 run length as a function of [ATP] and force. Unfilled circles are simulated data, while filled circles are experimental data taken from Milic et al. [16] and Andreasson et al. [17]. (**a**) Run length *versus* [ATP] at *F_x_* = +4 pN. (**b**) Run length *versus F_x_* at [ATP] = 2 mM.

Third, we study kinesin-1. The results simulated using λ = 1 were presented before [21]. Here, we use λ = 1.2 (see Table 1) to make simulations. Figure 7a,b shows simulated results of the run length *versus* [ATP] under a forward load (4 pN) and *versus* the applied load at saturating [ATP] (2 mM), respectively, where the corresponding single-molecule data of Milic et al. [16] and Andreasson et al. [17] are also plotted for comparisons. The simulated data are in quantitative agreement with the experimental data.

Now, we make comparisons of the results among the three kinesin dimers. First, we focus on the dependence of the run length upon [ATP]. From Figure 5a, Figure 6a and Figure 7a we see that the simulated run lengths for the three kinesin dimers are nearly independent of [ATP]. However, it is noted here that for simplicity of treatment, we have not taken into account the dissociation in the period of the ϕ- head binding strongly to MT. Taking into account the small dissociation probability in the *long* period when the MT-bound head in INT state is in ϕ state (Figure 1b) at low [ATP], the run length at low [ATP] should be slightly shorter than that at high [ATP], which is in better agreement with the single-molecule data [8,15,16,17].

Then, we turn to the run length *versus* the applied load. By comparing Figure 5b (for kinesin-5) with Figure 7b (for kinesin-1), it is interestingly seen that for kinesin-5 the run length is nearly symmetric with respect to the loading direction, and by contrast, for kinesin-1 the run length is dramatically asymmetric with respect to the loading direction, with the run length under a moderate backward load being much larger than that under a moderate forward load. The rather different features of the effect of loading direction on the run length between the two kinesin dimers can be explained as follows.

Since the moving time of the detached ADP-head between the MT-binding site and INT position is very short (in the order of 1–10 μs), the dissociation of the dimer from MT takes place mainly in INT state, where one head with undocked NL is bound to MT and the other ADP-head is bound strongly to the MT-bound head. Moreover, since the MT-bound head in ϕ, ATP or ADP.Pi state binds strongly to MT, its dissociation from MT is not considered. Hence, the dissociation of the dimer takes place mainly during two periods in INT state. One is the period after Pi is released from the MT-bound head and before the binding energy of the MT-bound ADP-head to MT changes from *E*_w1_ to *E*_w2_ (called Period I, see Figure 1f). In Period I, since *E*_w1_ is very small, the MT-bound head can easily dissociate from MT by overcoming *E*_w1_ within time *t*_r_. The other period is that the MT-bound head in INT state is in ADP state, with its binding energy to MT being *E*_w2_ (called Period II, see Figure 1g). In Period II, the MT-bound head can dissociate from MT with a large probability by overcoming *E*_w2_ before ADP is released within a time (1/*k*_D_) that is much longer than *t*_r_.

For kinesin-1, in INT state the NL-docking rate of the MT-bound head in ADP.Pi state, *k*_NL_ = 800 s^−1^ (see Table 1), is only 5-fold larger than its Pi-release rate, *k*_Pi_ = 160 s^−1^ (see Table 1), when the NL points forward. On the other hand, in INT state before the NL docking of the MT-bound head the NL is driven to point forward under the forward load. Thus, it has a large probability in each step for the Pi release to take place in INT state before the NL docking takes place under a forward load, giving a large probability for the occurrence of Period I. By contrast, under no or backward load with the NL of the MT-bound head being not pointed forward in INT state, since *k*_NL_ = 800 s^−1^ or $k_{\mathrm{NL}}/\lambda$ = 667 s^−1^ (λ = 1.2, see Table 1) is much (about 400-fold or 333-fold) larger than the Pi-release rate, $k_{\mathrm{Pi}}/\rho$ = 2 s^−1^ (*k*_Pi_ = 160 s^−1^ and ρ = 80, see Table 1), there is a very small probability for the occurrence of Period I. Thus, under a forward load the kinesin-1 dimer in INT state can dissociate from MT with a much larger probability than under no or the moderate backward load, giving the run length under a moderate forward load to be much smaller than that under no or the intermediate backward load, with the run length being dramatically asymmetric with respect to the loading direction.

For kinesin-5, in INT state the NL-docking rate of the MT-bound head in ADP.Pi state, *k*_NL_ = 300 s^−1^ (see Table 1), is 20-fold larger than its Pi-release rate, *k*_Pi_ = 15 s^−1^ (see Table 1), when the NL points forward. Thus, under a forward load there is a noticeable probability in each step for the Pi release to take place in INT state before the NL docking takes place, giving a noticeable probability for the occurrence of Period I. By contrast, under no or a backward load, with the NL of the MT-bound head in INT state being not pointed in the forward direction, *k*_NL_ = 300 s^−1^ or *k*_NL_/*λ* = 75 s^−1^ ( *λ* = 4, see Table 1) is much larger than the Pi-release rate *k*_Pi_/*ρ* = 0.19 s^−1^ (*k*_Pi_ = 15 s^−1^ and *ρ* = 80, see Table 1), giving a negligible probability for the occurrence of Period I. On the other hand, since the affinity of ADP-head to MT in Period II, *E*_w2_ = 36.5*k_B_T*, is relatively small, a large dissociation rate is expected in Period II. Thus, under no or the backward load the dissociation of the motor from MT arises almost solely from the dissociation taking place in Period II, while under the forward load the dissociation arises from both the dissociation taking place in Period II and the dissociation taking place in Period I. Due to the large dissociation rate in Period II, under the forward load the dissociation taking place in Period II would make a larger contribution to the overall dissociation than the dissociation taking place in Period I. It is noted that in Period II the dissociation rate is symmetric with respect to the direction of the applied load. On the other hand, the backward load decreases the velocity whereas the forward load has nearly no effect on the velocity (Figure 2b). By contrast, the forward load has an effect whereas the backward load has nearly no effect on the dissociation taking place in Period I. Consequently, a near symmetry of the run length with respect to the loading direction is expected.

From Figure 6b, it is seen that for kinesin-2, the run length under a forward load is also much smaller than that under no load, which is similar to the case of kinesin-1. As in the case of kinesin-1 (see above), this feature for kinesin-2 can also be explained as follows. In INT state, when the NL points forward under the forward load the NL-docking rate of the MT-bound head in ADP.Pi state, *k*_NL_ = 1200 s^−1^ (see Table 1), is only about 8-fold (KIF3A/A) and 6.7-fold (KIF3B/B) larger than the Pi-release rate, *k*_Pi_ = 150 s^−1^ for KIF3A/A and *k*_Pi_ = 170 s^−1^ for KIF3B/B (see Table 1). Thus, the dissociation taking place in Period I under the forward load has a large effect on the run length.

By comparing Figure 6b with Figure 5b and Figure 7b, it is interestingly seen that unlike kinesin-1 and kinesin-5, where run lengths are dependent only moderately on the backward load, there exist two regimes of processivity for kinesin-2: unloaded and loaded. Unloaded run lengths are long for kinesin-2, but even a small backward load can cause the run lengths to decrease greatly. The peculiar feature for kinesin-2 compared to kinesin-1 and kinesin-5 can be understood as follows.

For kinesin-1 and kinesin-5, under a backward load the NL-docking rates of the MT-bound heads in ADP.Pi state, *k*_NL_/*λ* = 667 s^−1^ (*k*_NL_ = 800 s^−1^ and *λ* = 1.2, see Table 1) and *k*_NL_/*λ* = 75 s^−1^ (*k*_NL_ = 300 s^−1^ and *λ* = 4, see Table 1), are much larger than the Pi-release rates, *k*_Pi_/*ρ* = 2 s^−1^ (*k*_Pi_ = 160 s^−1^ and *ρ* = 80, see Table 1) and *k*_Pi_/*ρ* = 0.19 s^−1^ (*k*_Pi_ = 15 s^−1^ and *ρ* = 80, see Table 1), respectively. Thus, the dissociation probabilities of the two motors from MT which arise from the dissociations taking place in period I are much smaller than those which arise from the dissociations taking place in period II, as in the case under no load. As a result, their run lengths are expected to depend only moderately upon the backward load. By contrast, for kinesin-2 KIF3A/A, under a backward load the NL-docking rate of the MT-bound head in ADP.Pi state, *k*_NL_/*λ* = 300 s^−1^ (*k*_NL_ = 1200 s^−1^ and *λ* = 4, see Table 1), is about 34-fold larger than the Pi-release rate, *k*_Pi_/*ρ* = 8.8 s^−1^ (*k*_Pi_ = 150 s^−1^ and *ρ* = 17, see Table 1). On the other hand, since the affinity of ADP-head to MT in Period II, *E*_w2_ = 40.8*k_B_T*, is large, a small dissociation rate is expected in Period II. Thus, the dissociation probability of kinesin-2 KIF3A/A from MT which arises from the dissociation taking place in period I has a large effect on the run length. By comparison, under no load the NL-docking rate of the MT-bound head in ADP.Pi state, *k*_NL_ = 1200 s^−1^ (see Table 1), is about 136-fold larger than the Pi-release rate, *k*_Pi_/*ρ* = 8.8 s^−1^ (*k*_Pi_ = 150 s^−1^ and ρ = 17, see Table 1). Hence, a backward load can cause a large reduction of the run length compared to that under no load.

It should be mentioned here that apart from the above explanation of the unloaded and loaded effects on the run length of kinesin-2, an additional possible explanation is stated as follows. For the unloaded case, in INT state an interaction could be present between the MT-bound head and coiled-coil segment, inhibiting Pi release (i.e., increasing ρ) in INT state and thus reducing the occurrence probability of Period I. By contrast, the load on the coiled-coil could disrupt this interaction between the MT-bound head and coiled-coil segment, with the Pi-release rate (i.e., with ρ) in INT state as given in Table 1. In this work, we have not considered this effect. The inclusion of the effect will further enlarge the difference between the unloaded and loaded run lengths but have little effect on the velocity because ρ is very large.

Taken together, although the three families of kinesin dimers show much similar mechanism of processive movement they show rather different features of the run length *versus* the external load. The rather different features arise mainly from the different values of the rate constants of the ATPase activity and NL docking.

### 3.3. Dynamics of Kinesin Dimers with Changed NL Lengths

**Figure 8:**
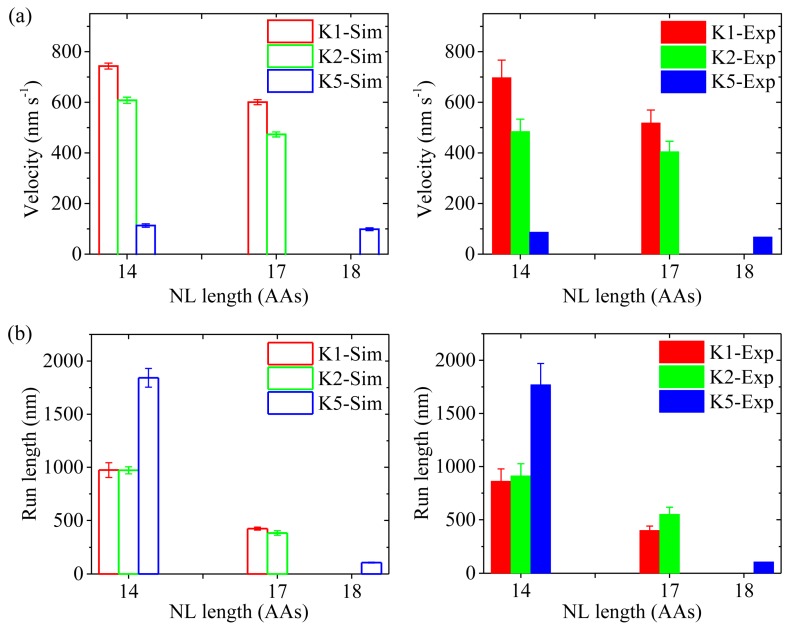
Effects of NL length on velocity and run length for kinesin-1, kinesin-2 KIF3A/B and kinesin-5 dimers under no load and saturating [ATP] (2 mM). Left panels are simulated data, while right panels are experimental data taken from Mickolajczyk and Hancock [19] for kinesin-1 and kinesin-2 KIF3A/B and from Shastry and Hancock [20] for kinesin-5. (**a**) Velocity *versus* number of residues in the NL of one head. (**b**) Run length *versus* NL length in one head.

First, we study kinesin-5 with the NL of each head being shortened to 14 AAs via deletion of the last four AAs (ΔKKAL to make Kin5_14_), as done in Shastry and Hancock [20]. Since for Kin5_14_ the internally stretched force on the NLs has a large value of about 33 pN in 2HB state (see Figure S4, at extension of 4.1 nm), the rates of ADP release from the leading and trailing heads are taken as $k_{D}^{\left(L\right)}=\sigma k_{D}$ and $k_{D}^{\left(T\right)}=k_{D}$ (*k*_D_ = 50 s^−1^, see Table 1), with σ = 7. In INT state, the rate of ADP release from the MT-bound head is also equal to *k*_D_. Since the four deleted AAs are not involved in the NL docking and are distant away from the motor domain, the deletion should have no effect on the interaction between the head and MT, interaction between the two heads, NL docking and ATPase activity except ADP release. Hence, we take the same values for all parameters for Kin5_14_ as those for the wild-type case (called Kin5_18_) except *k*_D_ and the force-extension relation of the NL (see Figure S4). In Figure 8a,b (left panels) we show simulated results of the velocity and run length, respectively, under no load at saturating [ATP] (2 mM) for Kin5_14_, and compare with the corresponding results for Kin5_18_. Interestingly, from Figure 8 we see that while the velocity for Kin5_14_ is almost unchanged relative to Kin5_18_, the run length for Kin5_14_ is increased significantly. These results on the dependence of run length and velocity upon length of NL are in good agreement with the experimental data of Shastry and Hancock [20] (right panels of Figure 8). The results can be explained as follows. Since for both Kin5_18_ and Kin5_14_ the rates of ADP release from the leading head are much larger than the rates of Pi release in the trailing head, their velocities are mainly determined by the rates of Pi release in the trailing head. Since the rates of Pi release in the trailing head for Kin5_18_ and Kin5_14_ are the same, their velocities are nearly the same. Since for Kin5_14_ the rate of ADP release from the leading head is much larger than that for Kin5_18_, the probability of Pi release taking place in the trailing head before ADP release taking place in the leading head for Kin5_14_ is much smaller than that for Kin5_18_, i.e., the occurrence probability of Period II for Kin5_14_ is much smaller than that for Kin5_18_. Thus, the run length for Kin5_14_ is much longer than that for Kin5_18_.

Second, we study kinesin-2 (KIF3A/B) with the NL of each head being shortened to 14 AAs by deleting the last three AAs (ΔDAL in KIF3A and KIF3B heads to make Kin2_14_), as done in Mickolajczyk and Hancock [19]. As in the case of Kin5_14_ (see above), for Kin2_14_ the rates of ADP release from the leading and trailing heads are taken as $k_{D}^{\left(L\right)}=\sigma k_{D}$ and $k_{D}^{\left(T\right)}=k_{D}$ (*k*_D_ = 90 s^−1^, see Table 1), with σ = 3. In INT state, the rate of ADP release from the MT-bound head is also equal to *k_D_*. In addition, we take the same values for all parameters for Kin2_14_ as those for the wild-type case (called Kin2_17_) except *k*_D_ and the force-extension relation of the NL (see Figure S5). In Figure 8a,b (left panels) we show simulated results of the velocity and run length, respectively, under no load at saturating [ATP] (2 mM) for Kin2_14_, and compare with the corresponding results for Kin2_17_. For comparison, the available single-molecule data [19] are shown in right panels of Figure 8. We see that the simulated and experimental data are in agreement with each other. The velocity for Kin2_14_ has a small increase relative to Kin2_17_ and the run length for Kin2_14_ has a large increase relative to Kin2_17_, which can be understood as follows. For Kin2_17_ the rate of ADP release from the leading head is smaller than the rate of Pi release in the trailing head whereas for Kin2_14_ the rate of ADP release from the leading head is larger than the rate of Pi release in the trailing head, resulting in the velocity for Kin2_17_ to be smaller than that for Kin2_14_. Since for Kin2_17_ the rate of ADP release from the leading head is smaller than that for Kin2_14_, the probability of Pi release in the trailing head taking place before ADP release taking place in the leading head for Kin2_17_ is larger than that for Kin2_14_, i.e., the occurrence probability of Period II for Kin2_17_ is larger than that that for Kin2_14_. Thus, the run length for Kin2_17_ is shorter than that for Kin2_14_. In addition, the simulated mean duration of one-head-bound (1HB) or INT state for Kin2_14_ (about 5.4 ms) is shorter than that for Kin2_17_ (about 8.6 ms) and the mean duration of 2HB state for Kin2_14_ (about 8.4 ms) is equal to that for Kin2_17_ (about 8.4 ms), which are also consistent with the single-molecule data [19].

Third, we study kinesin-1 with the NL of each head being extended to 17 AAs by inserting three AAs (DAL) into the C-terminal portion of the linker region (called Kin1_17_), as done in Mickolajczyk and Hancock [19]. Since for Kin1_17_ the internally stretched force on the NLs is small in 2HB state (see Figure S6, at extension of 4.1 nm), the rates of ADP release from both heads are taken to be the same, with $k_{D}^{\left(L\right)}=k_{D}^{\left(T\right)}=k_{D}/\sigma$ (*k*_D_ = 350 s^−1^ and σ = 2.3), as done for kinesin-5 and kinesin-2 (see above). In INT state, the rate of ADP release from the MT-bound head is also equal to $k_{D}/\sigma$. Since the added AAs in the NL are distant away from the motor domain, the addition should have no effect on the interaction between the head with MT, interaction between the two heads, NL docking and ATPase activity except ADP release. Thus, we take the same values for all parameters for Kin1_17_ as those for wild-type kinesin-1 (Kin1_14_) except *k*_D_ and the force-extension relation of the NLs (see Figure S6). 

In Figure 8a,b (left panels) we show simulated results of the velocity and run length, respectively, under no load at saturating [ATP] (2 mM) for Kin1_17_, where for comparison the corresponding simulated results for Kin1_14_ are re-shown. The available single-molecule data [19] are shown in right panels of Figure 8. We see that the simulated and experimental data are in agreement with each other. Both the velocity and run length for Kin1_17_ are reduced relative to those for Kin1_14_, with the velocity having a small reduction and the run length having a large reduction, which can be explained as follows. For Kin1_17_ with the nearly zero internally stretched force of the NLs, upon Pi release the trailing head cannot escape the potential well of depth *E*_w1_ = 19.8*k_B_T* (see Table 1) within time *t*_r_ with 100% probability, and thus more than one ATP molecules are hydrolyzed in the trailing head to make a forward step (noting that for kinesin-2 and kinesin-5, since *E*_w1_ ≤ 18*k_B_T*, even with nearly zero tension on the NLs, upon Pi release the trailing head can escape the potential well of depth *E*_w1_ within time *t*_r_ with nearly 100% probability). By contrast, for Kin1_14_, upon Pi release the large internally stretched force (about 30 pN) of the NLs can easily drive the trailing head to escape the potential well of depth *E*_w1_ within time *t*_r_ with 100% probability, and thus one ATP hydrolysis gives one forward step. Consequently, the velocity for Kin1_17_ is smaller than that for Kin1_14_. Since for Kin1_17_ the rate of ADP release from the leading head is smaller than that for Kin1_14_, the occurrence probability of Period II for Kin1_17_ is larger than for Kin1_14_. Thus, the run length for Kin1_17_ is shorter than that for Kin1_14_. In addition, the simulated mean duration of 1HB state for Kin1_14_ (about 3.2 ms) is shorter than that for Kin1_17_ (about 5.3 ms) and the mean duration of 2HB state for Kin1_14_ (about 7.8 ms) is close to that for Kin1_17_ (about 8.4 ms), which are also consistent with the experimental data [19].

Taken together, for the three families of kinesin dimers with longer NL lengths (17 or 18 AAs) the run lengths are reduced largely relative to those with shorter NL length (14 AAs). The reductions arise from the fact that the occurrence probability of INT state with the ADP-head bound to MT with the weak binding energy *E*_w2_ (i.e., the occurrence probability of Period II) is increased greatly, which is in turn due to the fact that the ADP-release rate in the leading head is decreased greatly because of the much reduced internally stretched force on the NLs.

### 3.4. Limping Effect of Kinesin Homodimers

Up to now, for simplicity of treatment, we have implicitly considered that the distance between the C-terminal end (i.e., the end connecting to the coiled-coil stalk) of one NL and that of another NL along the *x* direction (the movement direction) is zero. By considering a very small but non-zero distance along the *x* direction, which is called biased distance and is denoted by δ << *d*, and using model of Figure 1 but with the NL-docking rate *k*_NL_ → ∞ in ATP and ATP.Pi state, we showed that the asymmetric or limping movement dynamics of kinesin-1 homodimer can be explained well in our previous work [24].

In this section, we also consider a non-zero δ but finite values of *k*_NL_ to study the limping effect of homodimeric kinesin-1, kinesin-2 KIF3B/B and kinesin-5 with and without changes of NL lengths by using model of Figure 1 (considering a non-zero δ, the schematic of two successive forward steps of the homodimer at saturating [ATP] is shown in Figure S7). As discussed in detail before [24], the available evidence indicated that during the processive stepping of the kinesin dimer, the structure of the coiled-coil stalk is kept very stable, without unwinding [46], and moreover the stalk is kept in the fixed orientation [47,48]. Thus, to study limping we take the following into consideration. For odd steps (from Figure S7a to Figure S7c), before stepping the NL of each head in 2HB state is stretched to a length $r_{i}^{\left(O\right)}=d/2+\delta /2$ (Figure S7a) and after stepping the NL is stretched to a length $r_{f}^{\left(O\right)}=d/2-\delta /2$ (Figure S7c). For even steps (from Figure S7c to Figure S7e), before stepping the NL is stretched to a length $r_{i}^{\left(E\right)}=d/2-\delta /2$ (Figure S7c) and after stepping the NL is stretched to a length $r_{f}^{\left(E\right)}=d/2+\delta /2$ (Figure S7e). In this section, we focus on saturating [ATP] and take δ = 0.4 nm, which is much smaller than *d* = 8.2 nm, and values of all other parameter as given in Table 1.

**Figure 9:**
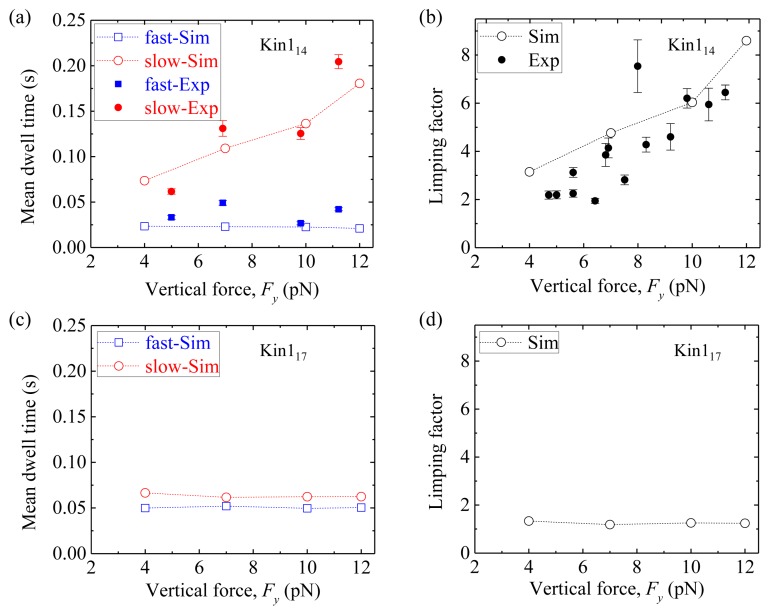
Limping effect of homodimeric kinesin-1 with and without extension of NLs. *F_x_* = –5 pN and δ = 0.4 nm in the simulation. (**a**) The mean dwell time of the odd (red circles) and even stepping phases (blue squares) *versus F_y_* for Kin1_14_. (**b**) Limping factor *versus F_y_* for Kin1_14_. In (**a**) and (**b**), unfilled circles and squares are simulated data, while filled circles and squares are experimental data taken from Asbury et al. [49] and Fehr et al. [50]. (**c**) The mean dwell time of the odd (red circles) and even stepping phases (blue squares) *versus F_y_* for Kin1_17_. (**d**) Limping factor *versus* vertical force *F_y_* for Kin1_17_.

First, we consider homodimeric kinesin-1. We fix longitudinal force *F_x_* = −5 pN and vary vertical force *F_y_*, as done in the single-molecule optical trapping experiments [49,50]. Figure 9a shows simulated results of the mean dwell time of the odd steps and that of the even steps *versus* vertical force *F_y_* for Kin1_14_, where for comparison the available single-molecule data [49,50] are also shown. We see that the mean dwell time of the odd steps are longer than the even steps, and the mean dwell time of the odd steps (also called slow steps) increases with *F_y_* whereas the mean dwell time of the even steps (also called fast steps) remains invariant. Figure 9b shows the results of the limping factor (defined as the ratio of the mean dwell time of the slow steps to that of the fast steps) calculated using data of Figure 9a, and compares with the experimental data [49,50]. 

We see that the limping factor for Kin1_14_ increases with *F_y_*. All the simulated results are consistent with the experimental data [49,50]. Figure 9c shows simulated results of the mean dwell time of the odd steps and that of the even steps *versus F_y_* for Kin1_17_. We see that both the mean dwell time of the odd steps and that of even steps are nearly independent of *F_y_* and both have the nearly same values, giving nearly no limping effect, with the limping factor nearly equal to one (Figure 9d).

**Figure 10:**
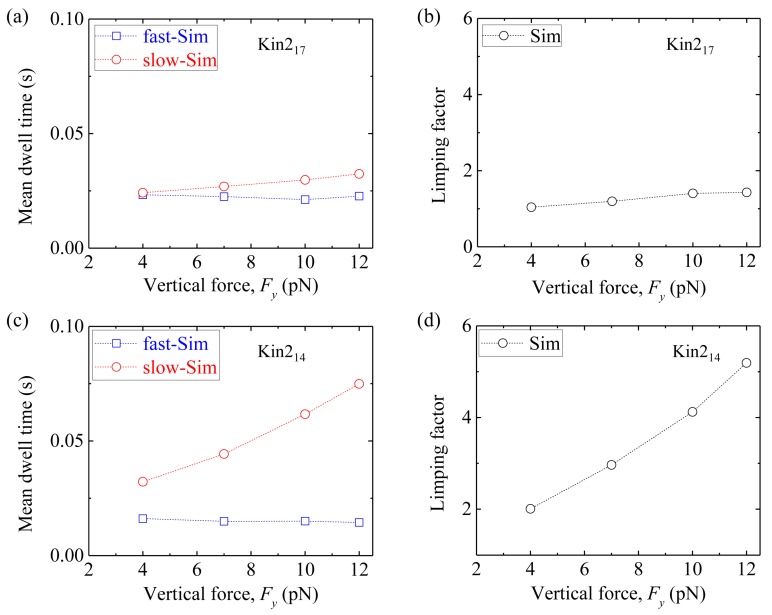
Limping effect of homodimeric kinesin-2 KIF3A/A with and without shortening of NLs. *F_x_* = −5.7 pN and δ = 0.4 nm in the simulation. (**a**) The mean dwell time of the odd (red circles) and even stepping phases (blue squares) *versus F_y_* for Kin2_17_. (**b**) Limping factor *versus F_y_* for Kin2_17_. (**c**) The mean dwell time of the odd (red circles) and even stepping phases (blue squares) *versus F_y_* for Kin2_14_. (**d**) Limping factor *versus F_y_* for Kin2_14_.

Second, we consider homodimeric KIF3B/B kinesin-2. We fix longitudinal force *F_x_* = −5.7 pN and vary vertical force *F_y_*. Figure 10a shows simulated results of the mean dwell time of the odd steps and that of the even steps *versus F_y_* for Kin2_17_. The corresponding results of the limping factor *versus F_y_* are shown in Figure 10b. It is seen that Kin2_17_ shows nearly no limping effect, as in the case of Kin1_17_. Figure 10c shows simulated results of the mean dwell time of the odd steps and that of the even steps *versus F_y_* for Kin2_14_. The corresponding results of the limping factor *versus F_y_* are shown in Figure 10d. As expected, Kin2_14_ shows similar limping effect to Kin1_14_.

**Figure 11:**
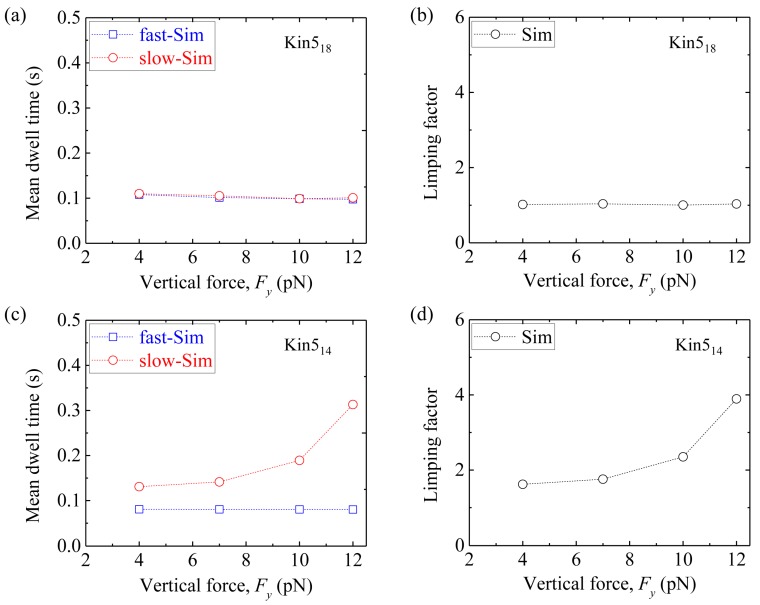
Limping effect of homodomeric kinesin-5 with and without shortening of NLs. *F_x_* = –3 pN and δ = 0.4 nm in the simulation. (**a**) The mean dwell time of the odd (red circles) and even stepping phases (blue squares) *versus F_y_* for Kin5_18_. (**b**) Limping factor *versus F_y_* for Kin5_18_. (**c**) The mean dwell time of the odd (red circles) and even stepping phases (blue squares) *versus F_y_* for Kin5_14_. (**d**) Limping factor *versus F_y_* for Kin5_14_.

Third, we consider homodimeric kinesin-5. We fix longitudinal force *F_x_* = −3 pN and vary vertical force *F_y_*. Figure 11a shows simulated results of the mean dwell time of the odd steps and that of the even steps *versus F_y_* for Kin5_18_. The corresponding results of the limping factor *versus F_y_* are shown in Figure 11b. As expected, Kin5_18_ shows no limping effect, as in the case of Kin1_17_ and Kin2_17_. Figure 11c shows simulated results of the mean dwell time of the odd steps and that of the even steps *versus F_y_* for Kin5_14_. The corresponding results of the limping factor *versus F_y_* are shown in Figure 11d. As expected, Kin5_14_ shows similar limping effect to Kin1_14_ and Kin2_14_.

Taken together, our simulated results show that while the wild-type kinesin-1 homodimer (Kin1_14_) shows the limping behavior, the kinesin-1 homodimer with extended NLs (Kin1_17_) does not show the limping behavior. By contrast, while the homodimeric kinesin-2 (Kin2_17_) and kinesin-5 (Kin5_18_) without change of the NLs do not show the limping behavior, the homodimeric kinesin-2 and kinesin-5 with shortened NLs (Kin2_14_ and Kin5_14_) show the limping behavior. While the simulated results on the limping dynamics of Kin1_14_ are consistent with the available single-molecule data [49,50], the predictions on the limping effect of Kin1_17_, Kin2_17_, Kin5_18_, Kin2_14_ and Kin5_14_ can be tested easily by using single-molecule optical trappings.

It is noted here that the consideration that during the processive stepping of the kinesin dimer its stalk is kept in the fixed orientation is not inconsistent with the recent single-molecule data of Ramaiya et al. [51]. This consideration implies that an intrinsic torque is present to keep the stalk in its equilibrium angle, where the line connecting the two C-terminus ends of NLs is parallel to the *x* axis. From the force-extension relation of the NL of the wild-type kinesin-1 head (Figure S1), it is seen that when the NL of the head is stretched to a length r=d/2+δ/2 = 4.3 nm the internally elastic force has a large value of about 53 pN, whereas when the NL is stretched to a length r=d/2−δ/2 = 3.9 nm, the internally elastic force is only about 15 pN. Thus, if the line connecting the two C-terminus ends of NLs is deviated slightly from that along the *x* axis, on average, a torque is present to make the coiled-coil stalk rotate in one direction during kinesin-1 processive movement at high [ATP], because a rotation angle relaxes to the equilibrium angle slowly with a timescale of 1 s [51]. Thus, accompanying the forward processive movement at high [ATP], the stalk would rotate gradually in one direction by overcoming the intrinsic torque until the torque caused by the stretching of the NL becomes equal to the intrinsic torque. However, at very low [ATP], a long time is present in INT state, during which the rotation induced by the torque caused by the stretching of the NL can be relaxed to its equilibrium angle. Thus, at very low [ATP], no rotation of the stalk would be observed, whereas at high [ATP] a rotation of small angle in one direction would be observed. Moreover, since the line connecting the two C-terminus ends of NLs can be deviated from the *x* axis in both directions with equal probability, both right- and left-handed rotations would be observed with equal probability. All these are in agreement with the single-molecule data of Ramaiya et al. [51]. It is noted that since the rotation angle is small (< 10°) [51], the rotation has little effect on the value of δ.

In addition, from the force-extension relation of the NL of the Kin1_17_ head (Figure S6), it is seen that when the NL of each head is stretched to a length r=d/2+δ/2 = 4.3 nm the internally elastic force is only about 11 pN, and when the NL is stretched to a length r=d/2−δ/2 = 3.9 nm the internally elastic force is only about 5 pN. Thus, it is predicted that for Kin1_17_, nearly no rotation of the stalk would be observed even at saturating [ATP]. Similarly, it is predicted that for a kinesin homodimer possessing no limping effect such as homodimeric kinesin-2 (Kin2_17_) and kinesin-5 (Kin5_18_), nearly no rotation of the stalk would be observed even at saturating [ATP]. By contrast, for a kinesin homodimer possessing pronounced limping effect, the rotation of small angle would be observed at high [ATP].

## 4. Conclusions

In this work, with our proposed model we give a consistent and quantitative explanation of the available single-molecule data on the dynamics such as the force dependence of velocity and processivity for three families of kinesins—*Drosophila* kinesin-1, kinesin-2 (mammalian KIF3A/B, KIF3A/A and KIF3B/B) and kinesin-5 (Eg5) dimers. The different experimental values of run length and velocity for different kinesins are reproduced by adjusting some parameters (e.g., *E*_w1_, *E*_w2_ and *E*_NL_) and with values of other parameters being chosen based on the available experimental data. The origin is revealed of kinesin-5 dimer exhibiting a near symmetry of the run length with respect to the direction of the applied load, kinesin-1 showing a dramatic asymmetry of the run length with respect to the direction of the applied load, while kinesin-2 dimers showing two (loaded and unloaded) regimes of processivity. The origin is revealed of the three dimers with shorter NL lengths having longer run lengths than with longer NL lengths.

Additionally, with our model the asymmetric or limping movement dynamics of the three kinesin-1, kinesin-2 and kinesin-5 homodimers with and without altered NL lengths are studied, providing predicted results (Figure 9, Figure 10 and Figure 11). The test of these predicted results by future experiments is critical not only to the understanding of the movement mechanism of kinesin dimers but also to the understanding of the origin of limping effect of the kinesin homodimers.

## Figures and Tables

**Table 1:** Values of parameters used in the simulation for wild-type kinesin-1, kinesin-2 and kinesin-5.

Parameter	Value			
	Kinesin-1	KIF3A	KIF3B	Kinesin-5
*E*_w1_	19.8*k_B_T*	≤18*k_B_T*	≤18*k_B_T*	≤15*k_B_T*
*E*_w2_	39.5*k_B_T*	40.8*k_B_T*	39.5*k_B_T*	36.5*k_B_T*
*E*_NL_	6*k_B_T*	9.3*k_B_T*	9.2*k_B_T*	7.5*k_B_T*
*k*_NL_ (s^−1^) (ADP.Pi state)	800	1200	1200	300
*k*_NL_ (s^−1^) (ATP state)	≤1	≤1.5	≤1.5	50
*k*_NL_ (s^−1^) (ADP state)	0	0	0	≤0.1
*k*_NL_ (s^−1^) (ϕ state)	0	0	0	≤0.1
*k*_NL_/*λ* (s^−1^) (under backward load)	*λ* = 1.2	*λ* = 4	*λ* = 2	*λ* = 4
*k*_Pi_ (s^−1^) (NL pointing forward)	160	150	170	15
*k*_Pi_/*ρ* (s^−1^) (NL not pointing forward)	*ρ* = 80	*ρ* = 17	*ρ* = 40	*ρ* = 80
*k*_b_ (μM·s^−1^)	2	2	2	0.5
*k*_H_ (s^−1^)	350	350	350	350
*k*_D_ (s^−1^) (leading head)	350	90	90	50
*k*_D_/*σ* (s^−1^) (trailing head)	*σ* = 2.3	*σ* = 1	*σ* = 1	*σ* = 1

## Supplementary Materials

The following are available online. Section S1: Potential of interaction between one kinesin head and MT in an ATPase cycle; Section S2: Potential characterizing the effect of NL docking of the MT-bound head on the movement of the tethered ADP-head; Section S3: Potential of interaction between two heads; Section S4: Interaction between the NL and head; Section S5: Equations for the mechanical stepping of the motor and its dissociation from MT; Section S6: Monte-Carlo simulations of processive movement and dissociation of the motor; Section S7: All-atom MD simulations of the force-extension relation of the NL; Figure S1: Force-extension relation of the NL of *Drosophila* kinesin-1 (Kin1_14_) head; Figure S2: Force-extension relations of the NL of KIF3A and KIF3B heads (Kin2_17_); Figure S3: Force-extension relation of the NL of kinesin-5 head (Kin5_18_); Figure S4: Force-extension relation of the NL of Kin5_14_ head; Figure S5: Force-extension relations of the NLs of Kin2_14_ heads; Figure S6: Force-extension relation of the NL of Kin1_17_ head; Figure S7: Model of asymmetric or limping stepping of kinesin-1, kinesin-2 and kinesin-5 homodimers with a non-zero distance (δ) between the C-terminus end of one NL and that of another NL at saturating ATP; Figure S8: Three typical simulated traces of displacement of the center-of-mass position of kinesin-5 dimer along the MT filament (*x* axis) *versus* time at 5 μM ATP and under no load.
