# Supplementary material for: Force Dependence of Velocity and Run Length of Kinesin-1, Kinesin-2 and Kinesin-5 Family Molecular Motors
Source: Molecules. 2019 Jan 14;24(2):287. doi: 10.3390/molecules24020287 (PMC6358798; doi:10.3390/molecules24020287)
Supplement: Supplementary file 1 [file molecules-24-00287-s001.pdf]

Supplementary Information

Force Dependence of Velocity and Run Length of Kinesin-1, Kinesin-2 and Kinesin-5 Family Molecular Motors

Si-Kao Guo [1,2], Wei-Chi Wang [1,2], Peng-Ye Wang [1,2,3] and Ping Xie [1,2,*]

1 Institute of Physics, Chinese Academy of Sciences, Beijing 100190, China; sikaoguo@gmail.com (S.-K.G.); weichi@aphy.iphy.ac.cn (W.-C.W.); pywang@aphy.iphy.ac.cn (P.-Y.W.)
2 School of Physical Sciences, University of Chinese Academy of Sciences, Beijing 100049, China
3 Songshan Lake Materials Laboratory, Dongguan, Guangdong 523808, China
* Correspondence: pxie@aphy.iphy.ac.cn; Tel.: +86-10-82649387, Fax: +86-10-82640224

Extended Materials and Methods

S1. Potential of interaction between one kinesin head and MT in an ATPase cycle

As mentioned in the main text, the available experimental data showed that the kinesin head in nucleotide-free, ATP or ADP.Pi state has a high binding energy to MT while in ADP state has a low binding energy [S1–S3]. Moreover, immediately after Pi release the head temporarily has a smaller binding energy to the local binding site (denoted by E_{w1}) on MT than to other unperturbed binding sites (denoted by $E_{w2} > E_{w1}$) and after time t_r, the binding energy of ADP-head to the local tubulin becoming E_{w2}, same as that to other tubulins. These changes of binding energy in an ATPase cycle can be explained as follows. In nucleotide-free, ATP or ADP.Pi state, the strong interaction between the head and local MT-tubulin induces conformational changes of the local tubulin [S4] (top panel inside box of Figure 1). After Pi release, the head changes rapidly its conformation from that in ADP.Pi form to that in ADP form, with the interaction between the ADP-head and MT-tubulin becoming weak. Since the weak interaction between the ADP-head and MT cannot induce conformational change of the MT-tubulin [S5], the deformed conformation of the local tubulin is then relaxed to the normally unchanged conformation in a time period of t_r. Thus, there exists a time period of t_r when the ADP-head has a very weak interaction with the local deformed tubulin with a binding energy E_{w1} (middle panel inside box of Figure 1). After t_r, the binding energy of the ADP-head to the local tubulin changes to E_{w2} (bottom panel inside box of Figure 1). After ADP release, the nucleotide-free head interacts strongly with MT and the strong interaction induces rapidly conformational changes of the MT-tubulin, with the potential returning to top panel inside box of Figure 1. Based on the above, the potential of the kinesin head interacting with MT in an ATPase cycle can be described mathematically as follows.

In nucleotide-free state the kinesin head binds strongly to MT, with the interaction potential being written as $V_S(x,y,z,\alpha,\theta,\phi) = V_{Sx}(x)V_y(y)V_z(z)V_\alpha(\alpha)V_\theta(\theta)V_\phi(\phi)$, where coordinate *oxyz* is defined in Figure 1, α, θ and ϕ are angles characterizing respectively the nutation, rotation and precession motions (when the kinesin head is in the MT-binding site, α, θ and ϕ correspond to the angles of rotations in *xoz*, *xoy* and *yoz* planes, respectively). Term $V_{Sx}(x) < 0$ (with the maxima equal to zero) represents the interaction potential between the kinesin head and MT along a MT protofilament and is approximately shown inside box of Figure 1. The available crystal structural data of the single kinesin head bound with MT showed that the head is bound sterically to the position between the α-tubulin and β-tubulin along an MT filament and not bound to the position between the β-tubulin and α-tubulin and other positions [S4,S6]. Thus, we take the period of $V_{Sx}(x)$, d = 8.2 nm, equal to the MT-tubulin

heterodimer repeat distance [S4,S6,S7]. Terms $V_y(y) \equiv \exp\left[-(y-y_0)/A_y\right]$ and $V_z(z) \equiv \exp\left(-|z-z_0|/A_z\right)$ denote the potential changes in the vertical and horizontal directions, respectively, with A_y and A_z characterizing the interaction distances. Note that due to the steric restriction of MT, the position of the kinesin head is confined to the region $y \geq y_0$. Terms $V_\alpha(\alpha) \equiv \exp\left(-|\alpha-\alpha_0|/A_\alpha\right)$, $V_\theta(\theta) \equiv \exp\left(-|\theta-\theta_0|/A_\theta\right)$ and $V_\phi(\phi) \equiv \exp\left(-|\phi-\phi_0|/A_\phi\right)$ denote the potential changes arising from the head rotations, with A_α, A_θ and A_ϕ characterizing the interaction angles. Here, we define $y_0 = z_0 = \alpha_0 = \theta_0 = \phi_0 = 0$ in the MT-binding state. These potential changes $V_y(y)$, $V_z(z)$, $V_\alpha(\alpha)$, $V_\theta(\theta)$ and $V_\phi(\phi)$ are similar to the Morse potential form that describes the van der Waals interaction. To be consistent with the Debye length that is in the order of 1 nm in solution, we take $A_y = A_z = 1$ nm and $r_{\text{head}}A_\alpha = r_{\text{head}}A_\theta = r_{\text{head}}A_\phi = 1$ nm, where the kinesin head is approximately a sphere of radius $r_{\text{head}} = 2.5$ nm. After ATP binding and then hydrolysis to ADP.Pi the kinesin head remains bound strongly to MT, with the interaction potential still being approximately described by $V_S(x,y,z,\alpha,\theta,\phi)$.

Immediately after Pi release, the interaction potential becomes one that can be written as $V_W(x,y,z,\alpha,\theta) = V_{Wx1}(x)V_y(y)V_z(z)V_\alpha(\alpha)V_\theta(\theta)V_\phi(\phi)$, with $V_{Wx1}(x) < 0$ being approximately shown in the middle panel inside box of Figure 1 and $V_y(y)$, $V_z(z)$, $V_\alpha(\alpha)$, $V_\phi(\phi)$ and $V_\phi(\phi)$ being the same as those defined above. Note that immediately after Pi release the binding affinity of kinesin for the local MT-binding site where the ADP.Pi-kinesin has just bound, E_{W1}, is smaller than at other MT-binding sites, E_{W2}. After a period of time, t_r, the affinity of the local MT-binding site for ADP-head relaxes to the normal value and the interaction potential becomes $V_W(x,y,z,\alpha,\theta) = V_{Wx2}(x)V_y(y)V_z(z)V_\alpha(\alpha)V_\theta(\theta)V_\phi(\phi)$, with $V_{Wx2}(x)$ being shown approximately in the bottom panel inside box of Figure 1. Note that the weak binding affinity of ADP-kinesin for MT, E_{W2}, is smaller than the strong binding affinity of nucleotide-free, ATP- or ADP.Pi-kinesin for MT, E_S.

Previous studies showed that for the case of monomeric kinesin such as KIF1A, asymmetric ratio d_1/d_2 of the potential (inside box of Figure 1) has a sensitive effect on the movement dynamics [S8–S10]. Here, we have checked that for the case of dimeric kinesin, the results for the movement dynamics (such as velocity and run length) are only determined sensitively by the depth of the weak potential well (E_{W1} and E_{W2}) while the form of the potential (whether it is asymmetric or symmetric) has little effect on the results. This is understandable, because in the case of dimeric kinesin, in order for the motor to make a forward step, it is only required that the trailing head is detach from site I while the other head is bound fixedly to site II (Figure 1a). Since the detachment involves only the head to escape from the potential well, it is expected the detachment is only sensitively determined by the depth of the potential well while is insensitive to the potential form. In the calculations we take the potential having the form as shown inside box of Figure 1, with the asymmetric ratio $d_1/d_2 = 3/5$, as done before for monomeric KIF1A [S10].

S2. Potential characterizing the effect of NL docking of the MT-bound head on the movement of the tethered ADP-head

In our model, the NL docking of the MT-bound head provides an energy barrier E_{NL} to prevent the tethered ADP-head from moving backward but allow the head to move forward freely. Thus, the effect of the NL docking on the motion of the tethered ADP-head relative to the MT-bound head can be approximately characterized by a potential $V_{NL}(x)$ having the form

$$V_{NL}(x) = E_{NL}, \quad x \leq 0, \tag{S1}$$
$$V_{NL}(x) = -E_{NL}(x-1), \quad 0 < x \leq 1, \tag{S2}$$
$$V_{NL}(x) = 0, \quad x > 1, \tag{S3}$$

where the MT-bound head is located at $(x, y, z) = (0, 0, 0)$.

S3. Potential of interaction between two heads

We take the potential of interaction between two kinesin heads with the NL of the MT-bound head being undocked having the following form:

$$V_{\mathrm{I}}(x,y,z,\alpha,\theta,\phi) = -E_{\mathrm{I1}} \exp\left(-\frac{\sqrt{(x-x_1)^2+(y-y_1)^2+(z-z_1)^2}}{A_{\mathrm{r}}}\right) \times \exp\left(-\frac{|\alpha-\alpha_1|}{A_\alpha}\right)\exp\left(-\frac{|\theta-\theta_1|}{A_\theta}\right)\exp\left(-\frac{|\phi-\phi_1|}{A_\phi}\right) \tag{S4}$$

where (x, y, z) is the center-of-mass coordinate of the detached ADP-head relative to that of the MT-bound head (which is taken as the origin of the coordinate) during one stepping period, (x_1,y_1,z_1) is the position of the detached ADP-head in the intermediate state, α_1, θ_1 and ϕ_1 are the nutation, rotation and precession angles of the detached ADP-head in the intermediate state, $E_{\mathrm{I1}} > 0$ is the strong interaction strength and A_{r} = 1 nm characterizes the interaction distance. Based on the available structural data [S11], we take $\alpha_1 = 180°$, $\theta_1 = -80°$ and ϕ_1 = 0 in the calculation, thus giving (x_1,y_1,z_1) = $2r_{\mathrm{head}} \times (\cos 80^\circ, \sin 80^\circ, 0)$. We have checked that taking other values of α_1, θ_1 and ϕ_1 has nearly no effect on our results. When the NL of the MT-bound head is docked, the potential is still described by Equation (S4), but with E_{I1} being replaced with E_{I2} (< E_{I1}).

S4. Interaction between the NL and head

To determine the interaction between the NL and kinesin head, we determine the elasticity of the linker by using all-atom MD simulations (see Section S6), as used elsewhere [S12]. Here, we take the NL of wild-type *Drosophila* kinesin-1 as an example to describe the simulation procedure. We take residues 324 through 338 from the structural data (PDB 3KIN), where residues 325 – 338 constitute the NL. We adjust the line connecting the alpha carbon (CA) atom of residue 324 and that of residue 338 along a given direction. We fix the CA atom of the residue 324 and impose a series of constant forces on the CA atom of the residue 338 along the given direction, as done before [S13]. We then calculate the distance, r_{NL}, between the two terminal CAs of the NL after reaching equilibrium, and a distance is calculated using data of 20-ns simulation after reaching equilibrium. In the literature, the calculated data of the force-extension relation of a flexible peptide were usually fitted by using the worm-like-chain (WLC) model. However, it is noted that the simulated data of the force-extension relation of the kinesin's NL are better fitted by using the exponential function than WLC model, especially at small values of pulling force (see Figure S1). Thus, here we use the exponential function to fit the simulated data

$$F_{\mathrm{NL}}(r_{\mathrm{NL}}) = a\exp(br_{\mathrm{NL}}) \tag{S5}$$

where a and b are constants. Then the force on the detached head, which results from the stretched NL, can be calculated by using Equation (S5).

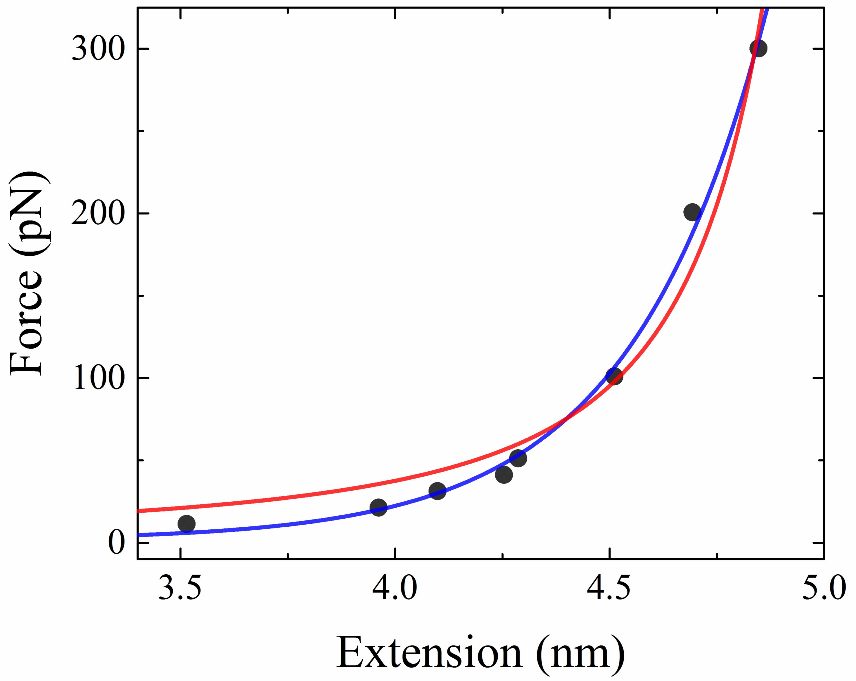

Figure S1. Force-extension relation of the NL of *Drosophila* kinesin-1 (Kin1$_{14}$) head. Dots represent the results obtained by using all-atom MD simulations. Red line is the fit curve with WLC model, $f(x) = k_B T / (4L_p) \left[(1 - x/L_c)^{-2} - 1 + 4x/L_c \right]$, where the contour length L_c = 5.26 nm and the persistence length L_p = 0.56 nm. Blue line is the fit curve with Equation (S5), $F_{NL}(r_{NL}) = a \exp(b r_{NL})$, with a = 7.064 × 10^{-5} pN and b = 3.148 nm^{-1}.

S5. Equations for the mechanical stepping of the motor and its dissociation from MT

In this work, for simplicity of analysis, we do not consider the dissociation of the nucleotide-free or ATP- or ADP.Pi-head from MT, because in nucleotide-free, ATP and ADP.Pi states the kinesin head binds to MT strongly. First, we present equations for the movement of the ADP-head relative to the nucleotide-free or ATP- or ADP.Pi-head bound fixedly to MT. We define the coordinate *oxyz* as shown in Figure 1, where the origin of the coordinate (0,0,0) is at the center-of-mass position of the MT-bound head. We consider the translation motion of the ADP-head in three dimensions (denoted by coordinates *x*, *y* and *z*) and rotation in three directions. The rotation is described by nutation motion (characterized by angle α), rotation motion (characterized by angle θ) and precession motion (characterized by angle ϕ). When the kinesin head is in the MT-binding site, α, θ and ϕ correspond to the angles of rotations in *xoz*, *xoy* and *yoz* planes, respectively.

As done in the single molecule optical trapping assays [S14–S16], we consider a bead with diameter of $2R_{bead}$ attached to the coiled-coil stalk of the dimer and an external force acting on the bead. With one kinesin head bound fixedly to MT at position (0,0,0), the translation and rotation of the other ADP-head relative to the MT-bound head in viscous solution can be described by Langevin equations [S17]:

$$\Gamma_x \frac{\partial x}{\partial t} = -\frac{\partial V_W(x,y,z,\alpha,\theta,\phi)}{\partial x} - \frac{\partial V_I(x,y,z,\alpha,\theta,\phi)}{\partial x} - \frac{\partial V_{NL}(x)}{\partial x} - F_{NL}\left(\frac{r}{2}\right)\frac{x}{r} + \xi_x(t)$$
$$\text{when } |x - x_{bead}| < |x_{bead}| \qquad \text{(S6)}$$

$$\Gamma_x \frac{\partial x}{\partial t} = -\frac{\partial V_{\mathrm{W}}(x,y,z,\alpha,\theta,\phi)}{\partial x} - \frac{\partial V_{\mathrm{I}}(x,y,z,\alpha,\theta,\phi)}{\partial x} - \frac{\partial V_{\mathrm{NL}}(x)}{\partial x} - F_{\mathrm{NL}}\left(\frac{r}{2}\right)\frac{x}{r}$$
$$- C\left[\left(x - x_{\mathrm{bead}}\right) - \mathrm{sgn}\left(x - x_{\mathrm{bead}}\right)\frac{d}{2}\right] H\left(\left|x - x_{\mathrm{bead}}\right| - \frac{d}{2}\right) + \xi_x(t)$$
$$\text{when } \left|x - x_{\mathrm{bead}}\right| \geq \left|x_{\mathrm{bead}}\right|, \tag{S7}$$

$$\Gamma_x^{(bead)} \frac{\partial x_{\mathrm{bead}}}{\partial t} = C\left[\left(0 - x_{\mathrm{bead}}\right) - \mathrm{sgn}\left(0 - x_{\mathrm{bead}}\right)\frac{d}{2}\right] H\left(\left|x_{\mathrm{bead}}\right| - \frac{d}{2}\right) + F_x + \xi_x^{(bead)}(t),$$
$$\text{when } \left|x - x_{\mathrm{bead}}\right| < \left|x_{\mathrm{bead}}\right|, \tag{S8}$$

$$\Gamma_x^{(bead)} \frac{\partial x_{\mathrm{bead}}}{\partial t} = C\left[\left(x - x_{\mathrm{bead}}\right) - \mathrm{sgn}\left(x - x_{\mathrm{bead}}\right)\frac{d}{2}\right] H\left(\left|x - x_{\mathrm{bead}}\right| - \frac{d}{2}\right) + F_x + \xi_x^{(bead)}(t),$$
$$\text{when } \left|x - x_{\mathrm{bead}}\right| \geq \left|x_{\mathrm{bead}}\right|, \tag{S9}$$

$$\Gamma_y \frac{\partial y}{\partial t} = -\frac{\partial V_{\mathrm{W}}(x,y,z,\alpha,\theta,\phi)}{\partial y} - \frac{\partial V_{\mathrm{I}}(x,y,z,\alpha,\theta,\phi)}{\partial y} - F_{\mathrm{NL}}\left(\frac{r}{2}\right)\frac{y}{r} + C\left(y_{\mathrm{bead}} - y\right) + \xi_y(t),$$
$$\text{when } y < 1 \text{ nm}, \tag{S10}$$

$$\Gamma_y \frac{\partial y}{\partial t} = -\frac{\partial V_{\mathrm{W}}(x,y,z,\alpha,\theta,\phi)}{\partial y} - \frac{\partial V_{\mathrm{I}}(x,y,z,\alpha,\theta,\phi)}{\partial y} - F_{\mathrm{NL}}\left(\frac{r}{2}\right)\frac{y}{r} + \xi_y(t),$$
$$\text{when } y \geq 1 \text{ nm}, \tag{S11}$$

$$\Gamma_y^{(bead)} \frac{\partial y_{\mathrm{bead}}}{\partial t} = C\left[\left(y - y_{\mathrm{bead}}\right) + \left(0 - y_{\mathrm{bead}}\right)\right] + F_y + \xi_y^{(bead)}(t), \quad \text{when } y < 1 \text{ nm}, \tag{S12}$$

$$\Gamma_y^{(bead)} \frac{\partial y_{\mathrm{bead}}}{\partial t} = C\left[\left(0 - y_{\mathrm{bead}}\right)\right] + F_y + \xi_y^{(bead)}(t), \quad \text{when } y \geq 1 \text{ nm}, \tag{S13}$$

$$\Gamma_z \frac{\partial z}{\partial t} = -\frac{\partial V_{\mathrm{W}}(x,y,z,\alpha,\theta,\phi)}{\partial z} - \frac{\partial V_{\mathrm{I}}(x,y,z,\alpha,\theta,\phi)}{\partial z} - F_{\mathrm{NL}}\left(\frac{r}{2}\right)\frac{z}{r} + \xi_z(t), \tag{S14}$$

$$\Gamma_\alpha \frac{\partial \alpha}{\partial t} = -\frac{\partial V_{\mathrm{W}}(x,y,z,\alpha,\theta,\phi)}{\partial \alpha} - \frac{\partial V_{\mathrm{I}}(x,y,z,\alpha,\theta,\phi)}{\partial \alpha} + \xi_\alpha(t), \tag{S15}$$

$$\Gamma_\theta \frac{\partial \theta}{\partial t} = -\frac{\partial V_{\mathrm{W}}(x,y,z,\alpha,\theta,\phi)}{\partial \theta} - \frac{\partial V_{\mathrm{I}}(x,y,z,\alpha,\theta,\phi)}{\partial \theta} + \xi_\theta(t), \tag{S16}$$

$$\Gamma_\phi \frac{\partial \phi}{\partial t} = -\frac{\partial V_{\mathrm{W}}(x,y,z,\alpha,\theta,\phi)}{\partial \phi} - \frac{\partial V_{\mathrm{I}}(x,y,z,\alpha,\theta,\phi)}{\partial \phi} + \xi_\phi(t), \tag{S17}$$

where $r = \sqrt{x^2 + y^2 + z^2}$, and x_{bead} and y_{bead} denote respectively x and y coordinates of the center-of-mass position of the bead (for simplicity but without loss of generality, the translation motion of the bead in the z coordinate is not considered here). $V_{\mathrm{W}}(x,y,z,\alpha,\theta,\phi)$ is the potential of the ADP-head interacting with MT-binding site during the period after Pi release and before ADP release with the other nucleotide-free or ATP- or ADP.Pi-head bound fixedly to MT (see Section S1), $V_{\mathrm{NL}}(x)$ is the potential characterizing the effect of the NL docking to the MT-bound head on the motion of the detached ADP-head (see Section S2), $V_{\mathrm{I}}(x,y,z,\alpha,\theta,\phi)$ is the potential of interaction between two kinesin heads (see Section S3), F_{NL} is the force acting on the ADP-head that results from the stretching of NLs (see Section S4), and d = 8.2 nm is the distance between two successive binding sites along a MT protofilament. For the longitudinal or x-component of the external force, F_x, the vertical or y-component is calculated by $F_y = |F_x| \tan\Theta_0$ and $\sin\Theta_0 = R_{\mathrm{bead}}/(R_{\mathrm{bead}} + l_{\mathrm{CC}})$, where l_{CC} = 54 nm is the length of the coiled-coil stalk of the kinesin dimer and $2R_{\mathrm{bead}}$ = 0.44 μm as used in the experiments [S14–S16]. F_x is defined as negative when it points backward (i.e., the $-x$ direction) and positive when it points forward (i.e., the $+x$ direction), while F_y is defined as positive when it points upward (i.e., the $+y$ direction). It is considered that the x-component of the interaction force between the bead and the kinesin dimer acts only on the NL of the head that has a larger distance to the bead along the x direction, and when the distance between a kinesin head and bead along the x direction is smaller than $d/2$ no internally elastic force exists between them.

Function sgn(x) is the sign function and function $H(x)$ is defined as follows: $H(x) = 1$ if $x > 0$ and $H(x) = 0$ if $x \leq 0$. It is noted that when the two heads are bound simultaneously to MT each head experiences a vertical force because the two heads have the same distance to the bead along the y direction, and when only one head is bound to MT and the other head is detached from MT only the MT-bound head experiences a vertical force because the distance of the bead to the MT-bound kinesin head along the vertical y direction is larger than that to the detached kinesin head. For approximation, we consider here that when the ADP-head is in the range of $0 \leq y < 1$ nm (when bound to MT the kinesin head is in the position of $y = 0$) the ADP-head experiences the vertical force, and when in other ranges ($y \geq 1$ nm) the ADP-head experiences no vertical force. The connection between the bead and C-terminus ends of the NLs is characterized by an elastic linear spring with a spring constant C. In the calculation we take $C = 0.1$ pN/nm (we have checked that varying value of C has little effect on the calculated results).

Due to the steric restriction of MT and considering the size of the kinesin head with radius $r_{\text{head}} = 2.5$ nm, it is required that $y \geq y_0 = 0$ and $r \geq 2r_{\text{head}} = 5$ nm. Due to the steric restriction of MT and considering the size of the bead with radius R_{bead}, it is required that $y_{\text{bead}} \geq R_{\text{bead}}$. The drag coefficients on the kinesin head are $\Gamma_x = \Gamma_y = \Gamma_z = 6\pi\eta_0 r_{\text{head}}$ and $\Gamma_\alpha = \Gamma_\theta = \Gamma_\phi = 8\pi\eta_0 r_{\text{head}}^3$, where η_0 is the solution viscosity in the vicinity of MT. Since the viscosity in the vicinity of MT is larger than that far away from MT, we take $\eta_0 = 0.02$ g cm^{-1} s^{-1} that is about 2-fold larger than that in water. $\xi_i(t)$ ($i = x, y, z, \alpha, \theta, \phi$) is the fluctuating Langevin force on the kinesin head, with $\langle \xi_i(t) \rangle = 0$, $\langle \xi_i(t)\xi_j(t') \rangle = 0$ ($i \neq j$) and $\langle \xi_i(t)\xi_i(t') \rangle = 2k_B T \Gamma_i \delta(t - t')$, where $k_B T$ is the thermal energy. The drag coefficients on the bead are $\Gamma_x^{(bead)} = \Gamma_y^{(bead)} = 6\pi\eta_0 R_{\text{bead}}$. Terms $\xi_x^{(bead)}(t)$ and $\xi_y^{(bead)}(t)$ are the fluctuating Langevin forces on the bead along the x and y directions, respectively, with $\langle \xi_j^{(bead)}(t) \rangle = 0$ ($j = x, y$), $\langle \xi_x^{(bead)}(t)\xi_y^{(bead)}(t) \rangle = 0$ and $\langle \xi_j^{(bead)}(t)\xi_j^{(bead)}(t') \rangle = 2k_B T \Gamma_j^{(bead)} \delta(t - t')$. The initial conditions for equations (S6) – (S17) are: $(x_0, y_0, z_0, \alpha_0, \theta_0, \phi_0) = (-d, 0, 0, 0, 0, 0)$, $x_{\text{bead0}} = -d/2 + F_x/C$, and $y_{\text{bead0}} = F_y/(2C)$.

Then, we present equations for the movement of kinesin when two heads are in ADP state. When both ADP-heads are bound simultaneously to MT, one head relative to the other head can still be described by equations (S6) – (S17). If one head is detached from MT with an affinity of E_{w1}, it would most probably bind immediately to other MT-bound ADP-head due to the high binding energy E_{I1} between them because the NL of the MT-bound ADP-head is undocked. If one head is detached from MT with an affinity of E_{w2}, it would either rebind immediately to MT or bind immediately to other MT-bound ADP-head. When the two ADP-heads are bound together strongly, the movement of the MT-bound ADP-head relative to MT can be described by the following equations [S17]:

$$\begin{aligned}\Gamma_x \frac{\partial x}{\partial t} = &-\frac{\partial V_{\text{W}}(x,y,z,\alpha,\theta,\phi)}{\partial x} + F_{\text{NL}}\left[\sqrt{(x_{\text{bead}}-x)^2+(y_{\text{bead}}-y)^2}-(R_{\text{bead}}+l_{\text{CC}})\right] \\ &\times H\left[\sqrt{(x_{\text{bead}}-x)^2+(y_{\text{bead}}-y)^2}-(R_{\text{bead}}+l_{\text{CC}})\right]\frac{x_{\text{bead}}-x}{\sqrt{(x_{\text{bead}}-x)^2+(y_{\text{bead}}-y)^2}}+\xi_x(t)\end{aligned}, \quad \text{(S18)}$$

$$\begin{aligned}\Gamma_x^{(bead)} \frac{\partial x_{bead}}{\partial t} = &-F_{\text{NL}}\left[\sqrt{(x_{\text{bead}}-x)^2+(y_{\text{bead}}-y)^2}-(R_{\text{bead}}+l_{\text{CC}})\right] \\ &\times H\left[\sqrt{(x_{\text{bead}}-x)^2+(y_{\text{bead}}-y)^2}-(R_{\text{bead}}+l_{\text{CC}})\right]\frac{x_{\text{bead}}-x}{\sqrt{(x_{\text{bead}}-x)^2+(y_{\text{bead}}-y)^2}}+F_x+\xi_x^{(bead)}(t)\end{aligned}, \quad \text{(S19)}$$

$$\begin{aligned}\Gamma_y \frac{\partial y}{\partial t} = &-\frac{\partial V_{\text{W}}(x,y,z,\alpha,\theta,\phi)}{\partial y} + F_{\text{NL}}\left[\sqrt{(x_{\text{bead}}-x)^2+(y_{\text{bead}}-y)^2}-(R_{\text{bead}}+l_{\text{CC}})\right] \\ &\times H\left[\sqrt{(x_{\text{bead}}-x)^2+(y_{\text{bead}}-y)^2}-(R_{\text{bead}}+l_{\text{CC}})\right]\frac{y_{\text{bead}}-y}{\sqrt{(x_{\text{bead}}-x)^2+(y_{\text{bead}}-y)^2}}+\xi_y(t)\end{aligned}, \quad \text{(S20)}$$

$$\Gamma_y^{(bead)} \frac{\partial y_{\text{bead}}}{\partial t} = -F_{\text{NL}} \left[\sqrt{(x_{\text{bead}} - x)^2 + (y_{\text{bead}} - y)^2} - (R_{\text{bead}} + l_{\text{CC}}) \right] \times H\left[\sqrt{(x_{\text{bead}} - x)^2 + (y_{\text{bead}} - y)^2} - (R_{\text{bead}} + l_{\text{CC}}) \right] \frac{y_{\text{bead}} - y}{\sqrt{(x_{\text{bead}} - x)^2 + (y_{\text{bead}} - y)^2}} + F_y + \xi_y^{(bead)}(t), \quad \text{(S21)}$$

$$\Gamma_z \frac{\partial z}{\partial t} = -\frac{\partial V_{\text{W}}(x, y, z, \alpha, \theta, \phi)}{\partial z} + \xi_z(t), \quad \text{(S22)}$$

$$\Gamma_\alpha \frac{\partial \alpha}{\partial t} = -\frac{\partial V_{\text{W}}(x, y, z, \alpha, \theta, \phi)}{\partial \alpha} + \xi_\alpha(t), \quad \text{(S23)}$$

$$\Gamma_\theta \frac{\partial \theta}{\partial t} = -\frac{\partial V_{\text{W}}(x, y, z, \alpha, \theta, \phi)}{\partial \theta} + \xi_\theta(t), \quad \text{(S24)}$$

$$\Gamma_\phi \frac{\partial \phi}{\partial t} = -\frac{\partial V_{\text{W}}(x, y, z, \alpha, \theta, \phi)}{\partial \phi} + \xi_\phi(t). \quad \text{(S25)}$$

where F_{NL} is the force acting on the MT-bound ADP-head that results from the stretched NLs (see Section S4). Here, for simplicity of treatment, the bead and C-terminus end of the NL of MT-bound ADP-head are implicitly considered to be rigidly connected. The initial conditions for Eqs. (S18) – (S25) are: ($x_0, y_0, z_0, \alpha_0, \theta_0, \phi_0$) = (0, 0, 0, 0, 0, 0), x_{bead0} = $(R_{\text{bead}} + l_{\text{CC}} + l_{\text{NL}}) F_x \big/ \sqrt{F_x^2 + F_y^2}$ (x_{bead0} = 0 when F_x = 0), and y_{bead0} = $(R_{\text{bead}} + l_{\text{CC}} + l_{\text{NL}}) F_y \big/ \sqrt{F_x^2 + F_y^2}$ ($y_{\text{bead0}} = R_{\text{bead}} + l_{\text{CC}}$ when $F_x = F_y = 0$), where l_{NL} is the length of the NL under a pulling force of magnitude $\sqrt{F_x^2 + F_y^2}$.

S6. Monte-Carlo simulations of processive movement and dissociation of the motor

Using equations (S6) – (S25) we can simulate the mechanical step of the movement of a kinesin head following Pi release relative to the other MT-bound kinesin head and the dissociation of the dimer from MT by using stochastic Runge–Kutta algorithm, as done before [S18,S19]. Then, we can simulate processive movement of the dimer by also considering continuous ATPase activities, which can be simulated using Monte Carlo algorithm, as used before [S19]. In the Monte Carlo simulations, during each time step Δt (Δt = 10^{-4} s in our simulation) a random number *ran* is generated with uniform probability between 0 and 1. The state transition with rate constant k_i (*i* = *T*, *H*, *Pi*, *NL*, *D*) takes place if *ran* $\leq P_i$ and the transition does not take place if *ran* > P_i. Here, $P_i = k_i \Delta t$ is the probability of state transition in each time step Δt, $k_T = k_b$[ATP] represents ATP binding rate to the nucleotide-free head, with k_b being the second-order rate constant for ATP binding and [ATP] being the ATP concentration, k_H represents the rate constant of ATP hydrolysis, k_{Pi} represents the rate constant of Pi release, k_{NL} represents the rate constant of NL docking into the motor domain of MT-bound head when the detached ADP-head is in the intermediate position, and k_D represents the rate constant of ADP releasing from ADP-head.

As mentioned above, we take the followings into consideration to study the dissociation of the dimer from MT. When one head of the dimer in nucleotide-free, ATP or ADP.Pi state binds strongly to MT, the binding affinity of the head to the MT is very large so that the dissociation of the dimer from the MT is negligible. This implies that only when two heads are simultaneously in ADP state the dissociation of the dimer is taken into account. In the calculations, when both heads move to positions of y > 10 nm, the dimer is considered to dissociate from MT.

In Figure S8, we show three typical simulated traces of displacement of the center-of-mass position of kinesin-5 dimer along the MT filament (x axis) *versus* time at 5 μM ATP and under no load. From each trace we can obtain the run length (the total displacement) and velocity (the total displacement being divided by the total time before dissociation) of the trace. The mean run length and velocity of a kinesin dimer at a given ATP concentration and a given load are computed using 1000 simulated traces.

S7. All-atom MD simulations of the force-extension relation of the NL

The all-atom MD simulations are carried out by using GROMACS5.1 [S20] with OPLS-AA/L all-atom force field [S21]. To avoid the edge effect, the distance between the peptide of the NL and the boundary of the box is at least 1.5 nm and much longer along the direction of the pulling force to stretch the NL. We add solvent and necessary ions with favorable concentration. Counter-ions are also added to neutralize the system. All MD simulations are run at 300K and 1 bar. The time step is set as 2 fs, and the output data is updated every ps. All chemical bonds are constrained using LINCS algorithm [S22]. The short-range electrostatics interaction and the cutoff for van der Waals interaction is set as 1 nm. Velocity-rescaling temperature coupling [S23] and Berendsen pressure coupling [S24] are used. The energy minimization is performed using the steepest descent method. Before the dynamic simulations, the systems are equilibrated successfully for 2 ns at 300 K and 1 bar pressure in the NVT ensemble and NPT ensemble, respectively. For calculations of the force-extension relation of the NL, after a 20-ns constant force-extension simulation, the distance between the two terminal CAs of the linker is extracted from the output trace files using the VMD1.9.2 [S25].

References

[S1] I. M. T. C. Crevel, A. Lockhart and R. A. Cross, Weak and strong states of kinesin and ncd. *Journal of Molecular Biology*, 1996, **257**, 66–76.

[S2] W. O. Hancock and J. Howard, Kinesin's processivity results from mechanical and chemical coordination between the ATP hydrolysis cycles of the two motor domains. *Proceedings of the National Academy of Sciences*, 1999, **96**, 13147–13152.

[S3] H. Sosa, E.J.G. Peterman, W.E. Moerner and L. S. B. Goldstein, ADP-induced rocking of the kinesin motor domain revealed by single-molecule fluorescence polarization microscopy. *Nature Structural Biology*, 2001, **8**, 540–544.

[S4] M. Morikawa, H. Yajima, R. Nitta, S. Inoue, T. Ogura, C. Sato and N. Hirokawa, X-ray and Cryo-EM structures reveal mutual conformational changes of Kinesin and GTP-state microtubules upon binding. *The EMBO Journal*, 2015, **34**, 1270–1286.

[S5] X.-X. Shi, Y.-B. Fu, S.-K. Guo, P.-Y. Wang, H. Chen and P. Xie, Investigating role of conformational changes of microtubule in regulating its binding affinity to kinesin by all-atom molecular dynamics simulation. *Proteins*, **2018**, *86*, 1127–1139.

[S6] L. A. Amos and D. Schlieper, Microtubules and maps. *Advances in Protein Chemistry*, 2005, **71**, 257–298.

[S7] H. Li, D.J. DeRosier, W.V. Nicholson, E. Nogales and K. H. Downing, Microtubule structure at 8 A resolution. *Structure,* 2002, **10**, 1317–1328.

[S8] R. D. Astumian and M. Bier, Fluctuation driven ratchets: Molecular motors, *Physical Review Letters*, 1994, **72**, 1766–1769.

[S9] P. Xie, Mechanism of processive movement of monomeric and dimeric kinesin molecules, *International Journal of Biological Sciences*, 2010, **6**, 665–674.

[S10] P. Xie, S.-X. Dou and P.-Y. Wang, Processivity of single-headed kinesin motors, *Biochimica et Biophysica Acta (BBA)-Bioenergetics*, 2007, **1767**, 1418–1427.

[S11] M. C. Alonso, D.R. Drummond, S. Kain, J. Hoeng, L. Amos and R. A. Cross, An ATP gate controls tubulin binding by the tethered head of kinesin-1, *Science*, 2007, **316**, 120–123.

[S12] Z.-W. Duan, P. Xie, W. Li and P.-Y. Wang, Are coiled-coils of dimeric kinesins unwound during their walking on microtubule? *PLoS ONE*, 2012, **7**, e36071.

[S13] V. Hariharan and W. O. Hancock, Insights into the mechanical properties of the kinesin

neck linker domain from sequence analysis and molecular dynamics simulations, *Cellular and Molecular Bioengineering*, 2009, **2**, 177–189.

[S14] J. O. Andreasson, S. Shastry, W.O. Hancock, and S. M. Block, The mechanochemical cycle of mammalian kinesin-2 KIF3A/B under load, *Current Biology*, 2015, **25**, 1166–1175.

[S15] B. Milic, J.O. Andreasson, W.O. Hancock, and S. M. Block, Kinesin processivity is gated by phosphate release, *Proceedings of the National Academy of Sciences*, 2014, **111**, 14136–14140.

[S16] J. O. Andreasson, B. Milic, G.Y. Chen, N.R. Guydosh, W.O. Hancock, and S. M. Block, Examining kinesin processivity within a general gating framework, *Elife*, 2015, **4**, e07403.

[S17] S.-K. Guo, X.-X. Shi, P.-Y. Wang, and P. Xie, Processivity of dimeric kinesin-1 molecular motors. *FEBS Open Bio*, **2018**, *8*, 1332–1351.

[S18] P. Xie, Stepping behavior of two-headed kinesin motors, *Biochimica et Biophysica Acta (BBA) – Bioenergetics*, 2008, **1777**: 1195-1202.

[S19] S.-K. Guo, P.-Y. Wang and P. Xie, A model of processive movement of dimeric kinesin, *Journal of Theoretical Biology*, 2017, **414**, 62–75.

[S20] M. J. Abraham, T. Murtola, R. Schulz, S. Páll, J.C. Smith, B. Hess and E. Lindahl, GROMACS: High performance molecular simulations through multi-level parallelism from laptops to supercomputers, *SoftwareX*, 2015, **1–2**, 19-25.

[S21] W. L. Jorgensen, D.S. Maxwell and J. Tirado-Rives, Development and testing of the OPLS all-atom force field on conformational energetics and properties of organic liquids, *Journal of the American Chemical Society*, 1996, **118**, 11225–11236.

[S22] B. Hess, P-LINCS: A parallel linear constraint solver for molecular simulation, *Journal of Chemical Theory and Computation*, 2008, **4**, 116–122.

[S23] G. Bussi, D. Donadio and M. Parrinello, Canonical sampling through velocity rescaling, *The Journal of Chemical Physics*, **2007**, *126*, 014101.

[S24] H. J. C. Berendsen, J.P.M. Postma, W.F. van Gunsteren, A. DiNola and J. R. Haak, Molecular dynamics with coupling to an external bath, *The Journal of Chemical Physics*, 1984, **81**, 3684–3690.

[S25] W. Humphrey, A. Dalke and K. Schulten, VMD-visual molecular dynamics, *Journal of Molecular Graphics*, 1996, **14**, 33–38.

.

SI figures.

.

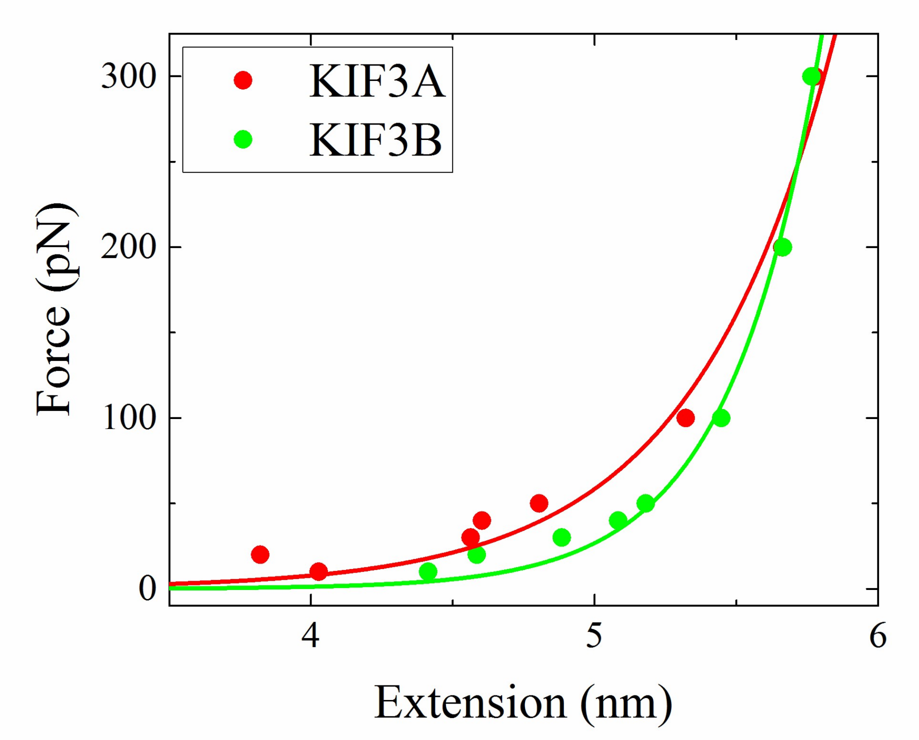

.

Figure S2. Force-extension relations of the NL of KIF3A and KIF3B heads (Kin2$_{17}$). Dots represent the results obtained by using all-atom MD simulations. Lines are the fit curves with Equation (S5), with a = 2.388×10^{-3} pN and b = 2.021 nm^{-1} for KIF3A head, and a = 4.647×10^{-6} pN and b = 3.113 nm^{-1} for KIF3B head.

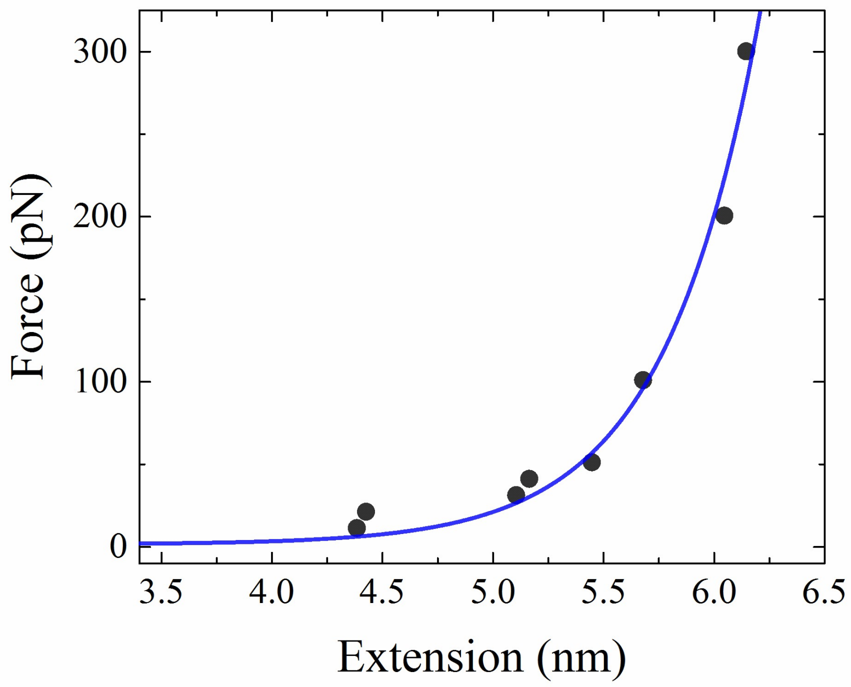

.

Figure S3. Force-extension relation of the NL of kinesin-5 head ($Kin5_{18}$). Dots represent the results obtained by using all-atom MD simulations. Line is the fit curve with Equation (S5), with $a = 1.900 \times 10^{-4}$ pN and $b = 2.307$ nm^{-1}.

.

.

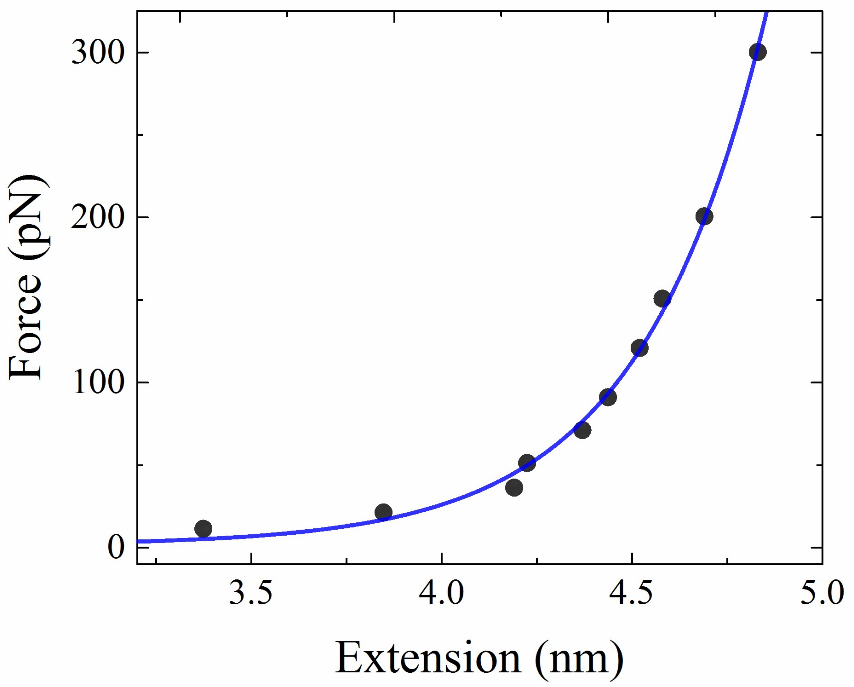

Figure S4. Force-extension relation of the NL of $Kin5_{14}$ head. Dots represent the results obtained by using all-atom MD simulations. Line is the fit curve with Eq. (S5), with $a = 1.438 \times 10^{-4}$ pN and $b = 3.010$ nm^{-1}.

.

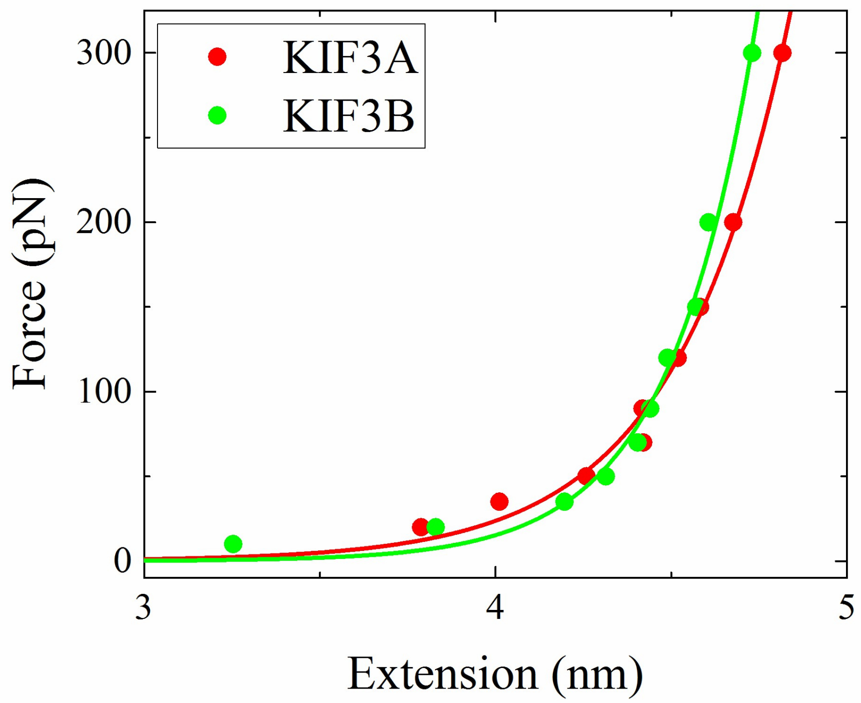

Figure S5. Force-extension relations of the NLs of Kin214 heads. Dots represent the results obtained by using all-atom MD simulations. Lines are the fit curves with Equation (S5), with $a = 9.247 \times 10^{-5}$ pN and $b = 3.114$ nm^{-1} for KIF3A head, and $a = 1.196 \times 10^{-6}$ pN and $b = 4.091$ nm^{-1} for KIF3B head.

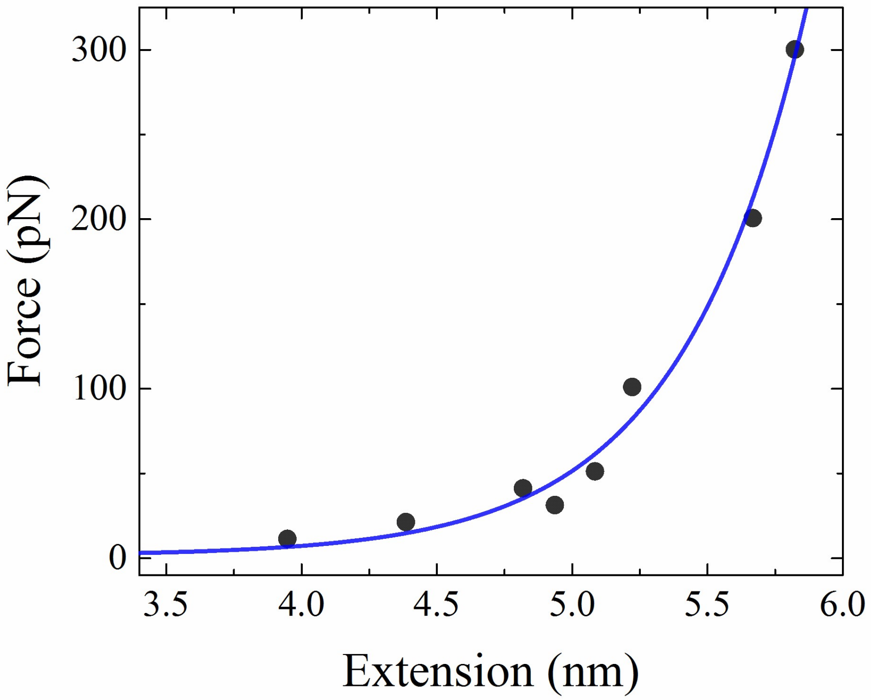

Figure S6. Force-extension relation of the NL of Kin1$_{17}$ head. Dots represent the results obtained by using all-atom MD simulations. Line is the fit curve with Equation (S5), with a = 1.060 × 10^{-3} pN and b = 2.150 nm^{-1}.

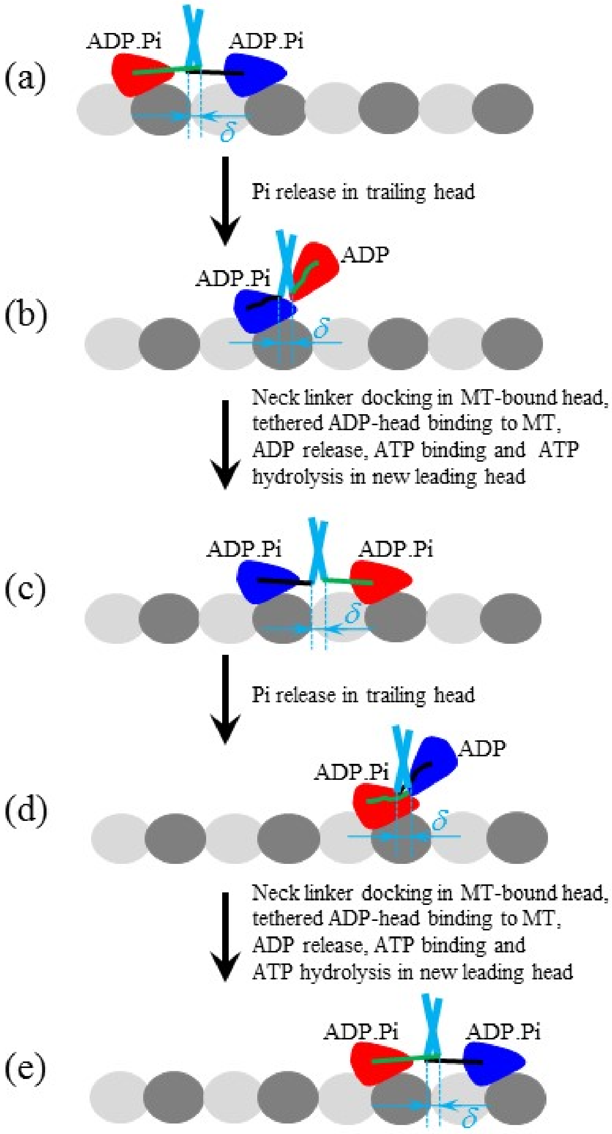

Figure S7. Model of asymmetric or limping stepping of kinesin-1, kinesin-2 and kinesin-5 homodimers with a non-zero distance (δ) between the C-terminus end of one NL and that of another NL at saturating ATP. For odd steps (from a to c), before stepping the NL of each head in 2HB state is stretched to a length $r_{\mathrm{i}}^{(O)} = d/2 + \delta/2$ (a) and after stepping the NL is stretched to a length $r_{\mathrm{f}}^{(O)} = d/2 - \delta/2$ (c). For even steps (from c to e), before stepping the NL is stretched to a length $r_{\mathrm{i}}^{(E)} = d/2 - \delta/2$ (c) and after stepping the NL is stretched to a length $r_{\mathrm{f}}^{(E)} = d/2 + \delta/2$ (e).

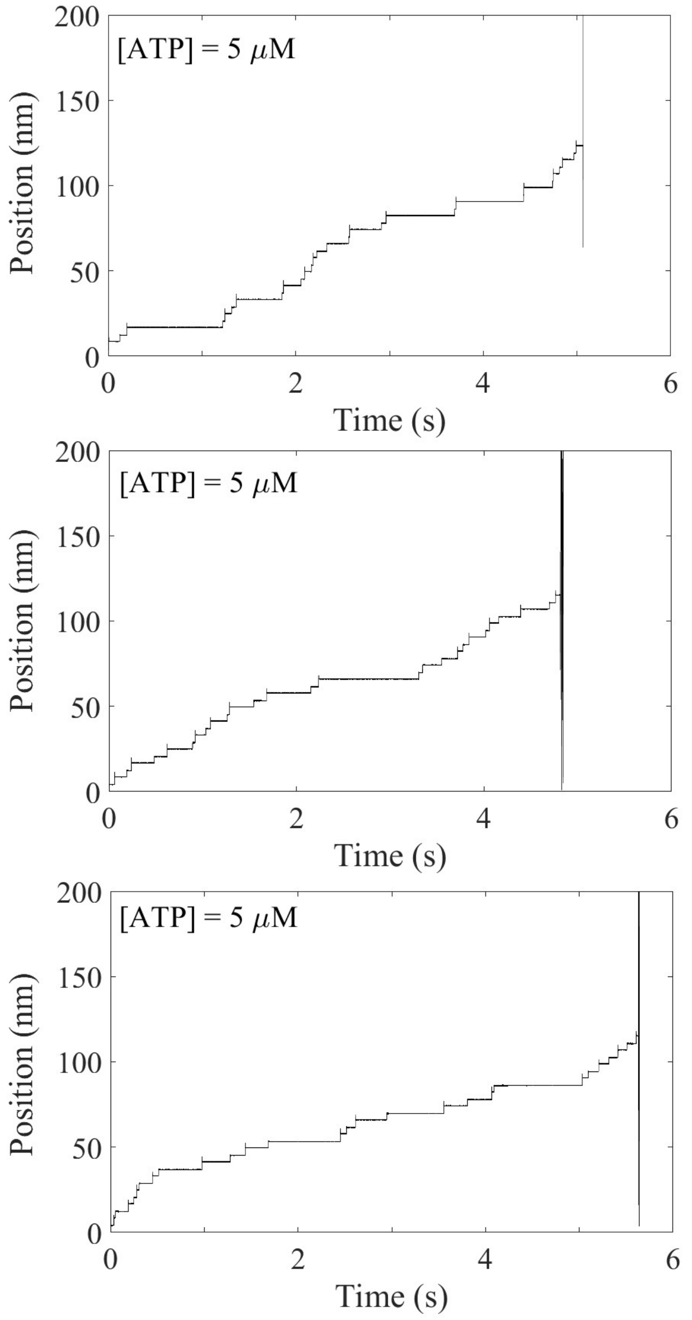

Figure S8. Three typical simulated traces of displacement of the center-of-mass position of kinesin-5 dimer along the MT filament (x axis) *versus* time at 5 μM ATP and under no load. Note that after dissociation from MT the position of the motor along the MT filament changes randomly.
